# Two newly established and mutually related subfamilies GH13_48 and GH13_49 of the α-amylase family GH13

**DOI:** 10.1007/s00253-024-13251-x

**Published:** 2024-07-11

**Authors:** Filip Mareček, Nicolas Terrapon, Štefan Janeček

**Affiliations:** 1 Laboratory of Protein Evolution, Institute of Molecular Biology, Slovak Academy of Sciences, 84551 Bratislava, Slovakia; 2 Architecture Et Fonction Des Macromolécules Biologiques, UMR CNRS, Aix-Marseille University, USC INRAE, 13288 Marseille, France; 3 Department of Biology, Institute of Biology and Biotechnology, Faculty of Natural Sciences, University of SS. Cyril and Methodius, 91701 Trnava, Slovakia

**Keywords:** Alpha-amylase family, GH13 subfamilies, Unique sequence features, Structural comparison, Evolutionary relatedness

## Abstract

**Abstract:**

Currently, the main α-amylase family GH13 has been divided into 47 subfamilies in CAZy, with new subfamilies regularly emerging. The present *in silico* study was performed to highlight the groups, represented by the maltogenic amylase from *Thermotoga neapolitana* and the α-amylase from *Haloarcula japonica*, which are worth of creating their own new GH13 subfamilies. This enlarges functional annotation and thus allows more precise prediction of the function of putative proteins. Interestingly, those two share certain sequence features, e.g. the highly conserved cysteine in the second conserved sequence region (CSR-II) directly preceding the catalytic nucleophile, or the well-preserved GQ character of the end of CSR-VII. On the other hand, the two groups bear also specific and highly conserved positions that distinguish them not only from each other but also from representatives of remaining GH13 subfamilies established so far. For the *T. neapolitana* maltogenic amylase group, it is the stretch of residues at the end of CSR-V highly conserved as L-[DN]. The *H. japonica* α-amylase group can be characterized by a highly conserved [WY]-[GA] sequence at the end of CSR-II. Other specific sequence features include an almost fully conserved aspartic acid located directly preceding the general acid/base in CSR-III or well-preserved glutamic acid in CSR-IV. The assumption that these two groups represent two mutually related, but simultaneously independent GH13 subfamilies has been supported by phylogenetic analysis as well as by comparison of tertiary structures. The main α-amylase family GH13 has thus been expanded by two novel subfamilies GH13_48 and GH13_49.

**Key points:**

*• In silico analysis of two groups of family GH13 members with characterized representatives*

*• Identification of certain common, but also some specific sequence features in seven CSRs*

*• Creation of two novel subfamilies—GH13_48 and GH13_49 within the CAZy database*

**Supplementary Information:**

The online version contains supplementary material available at 10.1007/s00253-024-13251-x.

## Introduction

The CAZy database (http://www.cazy.org/) classifies glycoside hydrolases (GHs) and other carbohydrate-active enzymes involved in the breakdown, biosynthesis and modification of carbohydrates into their sequence-based families (Drula et al. 2022). Starch is one of the most physiologically and economically significant polysaccharides on Earth, and enzymes involved in the degradation of starch and α-glucans related to starch are generally referred to as amylolytic enzymes (MacGregor et al. 2001). These enzymes belong to various GH families, e.g. GH13, GH14 and GH15 for α-amylase, β-amylase and glucoamylase, respectively, but also others (Janecek et al. 2014). The α-amylase (EC 3.2.1.1) is an integral part of the starch degradation process. It catalyzes the endohydrolysis of α-1,4-glucosidic linkages in polysaccharides that consist of at least three α-1,4-linked glucose units (MacGregor 1988). Until now, the α-amylase specificity, within the CAZy database, has been found in families GH13, GH57, GH119 and probably also in GH126 (Janecek et al. 2014; Janecek and Svensson 2022).

Currently (June 2024), the main α-amylase family GH13 contains more than 184,000 sequences with ~ 800 biochemically characterized members (Drula et al. 2022). This huge GH family covers enzymes with more than 30 different specificities belonging to three different enzyme classes: hydrolases (EC 3), transferases (EC 2) and isomerases (EC5) (Henrissat 1991; Takata et al. 1992; Jespersen et al. 1993; Janecek et al. 2014; Janecek and Svensson 2022). However, this family includes also some non-enzymatic representatives, i.e. heavy subunits of the heteromeric amino acid transporters rBAT and 4F2hc (Janecek et al. 1997; Gabrisko and Janecek 2009; Janecek and Gabrisko 2016; Fort et al. 2021). All family GH13 members should obey the following criteria (Matsuura et al. 1984; Kuriki and Imanaka 1999; Uitdehaag et al. 1999; MacGregor et al. 2001; Janecek 2002; van der Maarel et al. 2002; Janecek et al. 2014): (i) adopting a (β/α)_8_-barrel (i.e. the TIM-barrel) for the catalytic domain; (ii) employing the retaining reaction mechanism of α-glycosidic bond cleavage; (iii) sharing the catalytic machinery formed by the triad of acidic residues—aspartic acid (catalytic nucleophile), glutamic acid (donor proton) and aspartic acid (transition-state stabilizer) localized at the TIM-barrel strands β4, β5 and β7, respectively; and (iv) exhibiting four to seven conserved sequences regions (CSRs). However, these basic criteria may not strictly apply to all GH13 members, such as for the non-enzymatic members (rBAT and 4F2hc) mentioned above (Janecek and Gabrisko 2016; Fort et al. 2021).

In terms of domain organization, the family GH13 members typically contain three canonical domains: (i) the domain A—the catalytic TIM-barrel; (ii) the domain B—it protrudes out of the barrel connecting the strand β3 with the helix α3; and (iii) the domain C—succeeding the catalytic TIM-barrel (MacGregor 1993; Janecek 1994; Svensson 1994; Kuriki and Imanaka 1999; MacGregor et al. 2001). In addition to this three-domain canonical structure, these enzymes often consist of other non-catalytic modules, mainly the so-called starch-binding domains (SBDs) classified in CAZy as various carbohydrate-binding module (CBM) families (Janecek et al. 2019). In general, SBDs assist the catalytic domain through one or two binding sites to degrade starch and related substrates (Sorimachi et al. 1997; Janecek et al. 2019).

The α-amylase family GH13, at a higher level of hierarchy, forms with the families GH70 and GH77 the so-called clan GH-H (MacGregor et al. 2001; Janecek et al. 2014). Members of the clan GH-H—eventually with some slight modifications—share all the above-mentioned sequence-structural attributes of the family GH13. At a lower level of the hierarchy, the family GH13 has been divided into 47 subfamilies reflecting the fact that there are groups of enzymes which exhibit a higher degree of mutual sequence-structural similarity to each other than to the members of other groups (Stam et al. 2006). The family was originally divided into 35 GH13 subfamilies in 2006 (Stam et al. 2006), indicating that the creation of new subfamilies is an ongoing process (Cantarel et al. 2009). Here, the five most recently established GH13 subfamilies might be worth mentioning. The subfamily GH13_43 was created around the α-amylase from *Haloarcula hispanica* and other potential α-amylases from haloarchaeons (Janecek and Zamocka 2020), while the subfamily GH13_44 has brought together sequences represented by the α-glucosidase from unspecified Bifidobacteriaceae bacterium NR017 (Bhandari et al. 2021). The next subfamily GH13_45 might be of a special interest since it is formed by two subgroups of enzymes—the first one represented by the α-amylase BaqA from *Bacillus aquimari*s (Puspasari et al. 2013; Janecek et al. 2015), whereas the second one covers the amylolytic enzymes with a potentially aberrant catalytic triad as observed in the amylolytic enzyme BmaN1 from *Bacillus megaterium* (Sarian et al. 2017). The subfamily GH13_46 has been defined based on the *in silico* study focused on the cyclomaltodextrinase from *Flavobacterium* sp. No. 92 and other biochemically characterized amylolytic enzymes (Marecek and Janecek 2022). The most recently established subfamily GH13_47 contains the two α-1,6-glucosidic linkages tolerating α-amylases from *Bacteroides ovatus* (Brown et al. 2023) and *Rhodothermus marinus* (Miyasaka et al. 2024).

The main objective of the present study was to demonstrate that two related but still independent groups of GH13 sequences each deserves to appear as new subfamilies within the CAZy classification (Drula et al. 2022). The first group could be represented by amylolytic enzymes from *Thermotoga maritima* (Lim et al. 2003) and *Thermotoga neapolitana* (Park et al. 2010). While the pattern of reaction products of the *T. maritima* enzyme has not been published, the enzyme has nevertheless been designated as an α-amylase (Lim et al. 2003). On the other hand, the enzyme from *T. neapolitana* was shown to liberate maltose (together with a small amount of glucose) from soluble starch, amylose, amylopectin and glycogen, warranting to assign the enzyme the specificity of a maltogenic amylase (Park et al. 2010). For the *T. neapolitana* maltogenic amylase, also the three-dimensional structure has already been solved (Jun et al. 2013). Considering the high mutual sequence identity of the two above-mentioned enzymes, it is possible to assume that both enzymes represent maltogenic amylases. It is of note that this group includes additional characterized enzymes from: (i) a metagenomic-derived uncultured bacterium (Ariaeenejad et al. 2021); (ii) *Lactoplantibacillus plantarum* WCFS1 (Plaza-Vinuesa et al. 2019); and (iii) *Lactoplantibacillus plantarum* ST-III (Jeon et al. 2016). The tertiary structure of the first of the three proteins is available in the Protein Data Bank (PDB; Burley et al. 2021) under the PDB code 3DHU since 2008, but without associated publication. The second group could be established around the amylolytic enzyme from *Haloarcula japonica* that was biochemically characterized as an α-amylase 10 years ago (Onodera et al. 2013). This halophilic enzyme was found to be active mainly towards amylose, soluble starch and amylopectin, but also with a lower activity against glycogen (Onodera et al. 2013). Another close homologue, a halotolerant α-amylase, has recently been characterized in *Haloferax alexandrinus* WSP1 (Verma et al. 2020). Each of the two groups briefly described above exhibits their own specific sequence-structural features that clearly distinguish them from each other. However, since some of those features are shared between them, it has been demonstrated these groups are independent, but mutually related GH13 subfamilies, i.e. the *Thermotoga*-like and *Haloarcula*-like groups, providing increased functional annotation to their members.

## Materials and methods

### Sequence collection

At the beginning of the present study, seven members of the family GH13, yet not assigned to any subfamily in the CAZy classification (Drula et al. 2022; http://www.cazy.org/; update of 18 March 2024), were identified based on biochemical evidence of their activity in the literature as well as on the similarity of their catalytic domain: (i) ACF75909.1 from *Thermotoga neapolitana* (Park et al. 2010; Jun et al. 2013); (ii) AAD36717.1 from *Thermotoga maritima* (Lim et al. 2003); (iii) CAD62849.1 from *Lactoplantibacillus plantarum* WCFS1 (Plaza-Vinuesa et al. 2019); (iv) ADN97370.1 from *Lactoplantibacillus plantarum* ST-III (Jeon et al. 2016); (v) QYD13596.1 from the metagenomic-derived uncultured bacterium (Ariaeenejad et al. 2021); (vi) BAM75337.1 from *Haloarcula japon*ica (Onodera et al. 2013); and (vii) QIB80089.1 from *Haloferax alexandrinus* (Verma et al. 2020). Pairwise amino-acid sequence alignments, using BLASTp web-interface (Altschul et al. 1990; https://blast.ncbi.nlm.nih.gov/Blast.cgi), produced by querying all seven proteins, suggested two distinct groups: the α-amylases from *H. japonica* and *H. alexandrinus* (i.e. the *Haloarcula*-like) that exhibit only a more remote homology to remaining five enzymes (i.e. the *Thermotoga*-like) displaying a higher similarity levels.

Subsequently, since the sequences of maltogenic amylase from both *L. plantarum* strains are identical, homologous proteins were retrieved by PSI-BLAST (Altschul et al. 1997) searches using all six distinct protein sequences as queries against the NCBI nr (non-redundant) dataset with default parameters (run on the 12 December 2023). For all collected homologous proteins, their assignment to the GH13 family or to one of its subfamilies was extracted from the CAZy database. Based on the presence of sequences belonging to already established GH13 subfamilies within the individual PSI-BLAST searches, the E-value thresholds for membership in the new groups were estimated to 1e^−36^ and 1e^−53^ for the five *Thermotoga*-like and *Haloarcula*-like enzymes, respectively. As a result, 6325 (*Thermotoga*-like) and 802 (*Haloarcula*-like) non-redundant sequences were obtained, i.e. 7127 sequences in total. To reduce to a more manageable number of sequences, without losing diversity signals, the software UCLUST (Edgar 2010) with a sequence identity threshold value of 50% for both groups was used. At this point, the incomplete sequences were removed, resulting in a reduced set of 316 *Thermotoga*-like and 42 *Haloarcula*-like sequences, summing up to 364 sequences, with the six distinct characterized sequences aforementioned. After a preliminary alignment and phylogenetic analysis, 17 sequences from the 364 sample were removed as they did not contain the GH13 complete catalytic machinery and are likely resulting from sequencing errors or ongoing pseudogenization. In order to place this representative set of 347 obtained sequences into the overall family GH13 context, it was completed by 141 sequences classified in the 47 GH13 subfamilies established so far, i.e. three sequences from each subfamily. Those sequences were selected mainly with respect to available literature—especially, GH13 subfamily members with a solid biochemical characterization and/or available tertiary structure were prioritized. The final set thus consisted of 488 studied sequences (Table S1), which were retrieved from the UniProt (UniProt Consortium 2021; https://www.uniprot.org/) or GenBank (Sayers et al. 2021; https://www.ncbi.nlm.nih.gov/genbank/) databases.

### Sequence comparison and phylogenetic analysis

The multiple sequence alignments were performed using Clustal-Omega web-interface (Sievers et al. 2011; https://www.ebi.ac.uk/Tools/msa/clustalo/) with default parameters. First, the full set of 488 sequences (Table S1) was aligned, and the alignment was trimmed to cover the substantial part of the catalytic TIM-barrel domain including domain B, i.e. from the beginning of the CSR-VI (the strand β2) to the end of the CSR-VII (the strand β8). Information about the boundaries of the individual domains and other sequence-structural details were obtained from the literature and previous bioinformatics studies (MacGregor and Svensson 1989; Jespersen et al. 1991, 1993; Janecek et al. 1999; Kim et al. 1999; Janecek 2002; Oslancova and Janecek 2002; Hondoh et al. 2003; Lim et al. 2003; Abe et al. 2004; Tan et al. 2008; Koropatkin and Smith 2010; Park et al. 2010; Jun et al. 2013; Majzlova et al. 2013; Onodera et al. 2013; Puspasari et al. 2013; Peng et al. 2014; Xu et al. 2014; Janecek et al. 2015; Sarian et al. 2017; Janecek and Zamocka 2020; Marecek and Janecek 2022). To maximize the similarities, manual tuning was performed, especially within the CSRs. Then, after analysis of the resulting phylogenetic tree (based on all 488 sequences; described hereafter), a reduced sample of 38 sequences was prepared. In particular, the effort was to focus on the two potential new GH13 subfamilies, selecting representatives, if possible, from all three taxonomic kingdoms, as well as on one closely related GH13 group (eventual future GH13 subfamily) still awaiting a biochemical characterization, and their closest relatives. The reduced dataset thus consisted of 38 sequences as follows (Table S1): (i) 22 sequences from the *Thermotoga*-like subfamily (including four characterized enzymes); (ii) 11 sequences from the *Haloarcula*-like subfamily (including two characterized enzymes); (iii) two sequences of the potential future subfamily requiring a biochemical analysis; and (iv) three sequences from the subfamily GH13_38, which is the most closely related to the two novel subfamilies of the *Thermotoga*-like and *Haloarcula*-like groups. The multiple-sequence alignment of the reduced dataset was performed using the complete full-length sequences.

Based on the above-mentioned alignments, two evolutionary trees were constructed. Both were calculated using the maximum-likelihood reconstruction method (including the gaps in the alignments) with the LG substitution model (Le and Gascuel 2008) and the bootstrapping procedure (Felsenstein 1985) with 500 bootstrap trials implemented in the MEGA X package (Kumar et al. 2018). The trees were displayed using the iTOL programme (Letunic and Bork 2007; https://itol.embl.de/).

In order to support the creation of the two new GH13 subfamilies observed by sequence comparison and phylogeny, the program HMMER3 (Eddy 2011; http://hmmer.org/) was used to generate the hidden Markov model (HMM) for each of the two newly proposed *Thermotoga*-like and *Haloarcula*-like groups. HMMs provide higher discrimination levels than simple pairwise alignments, as these probabilistic models of multiple sequence alignments capture the (sub)family evolutionary fingerprints, that is, which positions have been more constrained, or conversely more relaxed.

Sequence logos of seven well-established CSRs were prepared by the WebLogo server (Crooks et al. 2004; http://weblogo.threeplusone.com/) for each of the two novel subfamilies of the *Thermotoga*-like and *Haloarcula*-like groups, and, for comparison, also for the subfamily GH13_38. The GH13_38 logo was prepared in the same way as that for the two novel subfamilies, i.e. the software UCLUST (Edgar 2010) with a sequence identity threshold value of 50% was applied to the 1294 sequences classified in the CAZy database (update of 18 March 2024).

### Comparison of tertiary structures

Three-dimensional structures were retrieved from PDB (Burley et al. 2021; https://www.rcsb.org/) for: (i) maltogenic amylase from *Thermotoga neapolitana* (PDB code: 4GKL; Jun et al. 2013); (ii) maltogenic amylase from *Lactoplantibacillus plantarum* (PDB code: 3DHU; unpublished); and (iii) one representative for each of 47 GH13 subfamilies established so far (Table S2). When no experimental three-dimensional structure was available for a given GH13 subfamily in PDB, the AlphaFold-generated model structure (Varadi et al. 2022; https://alphafold.ebi.ac.uk/) was used. This was also the case of the main representative of the *Haloarcula*-like subfamily, i.e. the α-amylase from *H. japonica*.

In all cases, only the three GH13 canonical domains (i.e. A + B + C domains) were superimposed, and the information on domain boundaries was obtained from published literature and available databases. All structural comparisons were performed using the 1.16 version of the UCSF Chimera program (Pettersen et al. 2004), which has also been used for visualization of structures.

## Results

### Evolutionary relationships in the GH13 family context

**Fig. 1:**
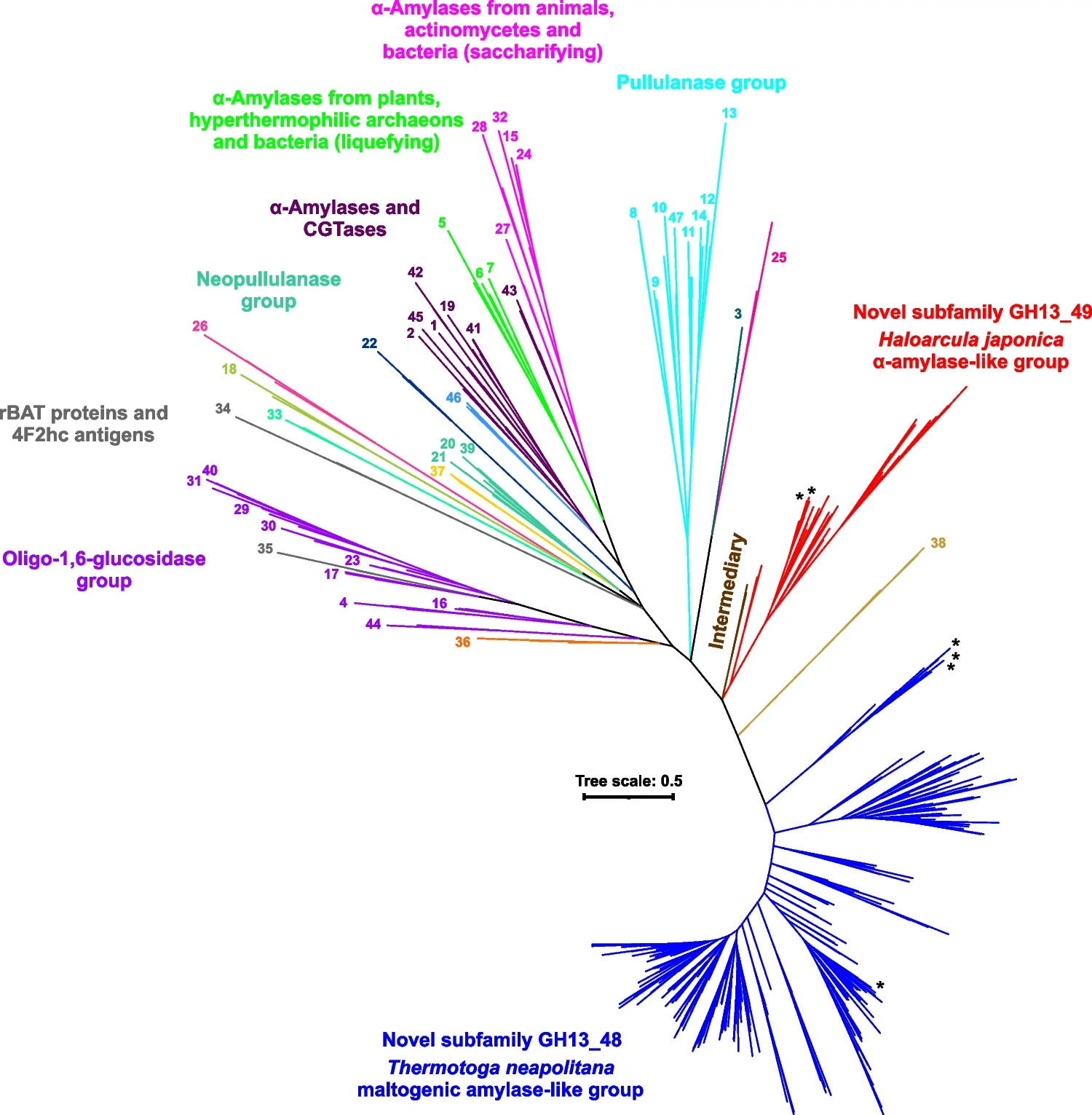
Evolutionary tree reflecting the relationships within the main α-amylase family GH13. The tree covers 488 sequences with a focus on the two novel closely related subfamilies GH13_48 and GH13_49 represented by the maltogenic amylase from *T. neapolitana* and the α-amylase from *H. japonica*, respectively (for details, see Table S1). The tree is based on the alignment (Fig. S1), spanning the sequence segment from the beginning of the strand β2 (CSR-VI) to the end of the strand β8 (CSR-VII), i.e. the substantial part of the catalytic TIM-barrel including the domain B. For the sake of simplicity, only the branches leading to the individual GH13 subfamilies, marked by their numbers, are shown. Characterized members of the two novel subfamilies are labelled by an asterisk. The same tree in the full version with all the leaves, i.e. with sequence description, is presented in Figure S2

Several characterized members of the family GH13, currently not assigned to any subfamily, were used as a starting point to gather a diverse set of 347 closely related homologous sequences. In order to indicate their mutual relationships, as well as their position in the context of the entire α-amylase family GH13, this dataset was completed with representative sequences of all 47 GH13 subfamilies (3 sequences for each) established so far, and its multiple sequence alignment was performed (Fig. S1). Since the full-length sequences of the many GH13 subfamilies are too diverse and their domain architecture—beyond the canonical three-domain arrangement—is highly variable, the alignment of full-length sequences contained an excessive background noise especially at the N- and C-termini of the catalytic module. In order to maximize the phylogenetic signal while reducing the drift, the alignment (Fig. S1) was thus trimmed to the segment that covers the most significant part of the GH13 catalytic domain, i.e. the sequence portion spanning the segment from the beginning of CSR-VI (the strand β2 of the catalytic TIM-barrel) to the end of CSR-VII (the strand β8 of the catalytic TIM-barrel) including the complete domain B. Based on the trimmed alignment, a maximum-likelihood evolutionary tree was calculated (Fig. 1), where most of the already established subfamilies formed an outgroup to the 347-sequence set, with the exception of the subfamily GH13_38. The 347-sequence set divided into three groups: (i) the *Thermotoga*-like—304 sequences represented by the maltogenic amylase from *T. neapolitana* (Park et al. 2010); (ii) the *Haloarcula*-like—41 sequences formed around the α-amylase from *H. japonica* (Onodera et al. 2013); and (iii) a small group with, currently, only two sequences still lacking any experimental characterization. The GH13_38 obviously forms, with the three above-mentioned groups of the dataset, a monophyletic clade.

The first group gathers five enzymes reported as maltogenic amylase or α-amylase in the literature and presents an important taxonomic diversity, covering various bacterial phyla, Bacteroidota and Bacillota being dominant, as well as a few from both Archaea and Eukaryota. These species span various environmental niches, such as gut, land, or aquatic (Chen et al. 2023), the latter exhibiting fusions, in a few dozen proteins, with SBDs of the family CBM26. The second group covers two proteins reported as α-amylase in the literature and is almost restricted to the archaeal kingdom, despite its large size/sequence diversity. Representatives of this group are almost exclusively extremophiles that inhabit hypersaline environments, such as saline and soda lakes, hypersaline soil, or marine solar saltern. Three bacterial sequences belonging to this group have been isolated near deep-sea hydrothermal vents (Chen et al. 2023). The third group consisting of only two bacterial sequences, may in the future, after the appropriate biochemical characterization of the member(s), define a novel GH13 subfamily. It should be pointed out here that the tree shown in Fig. 1 is a simplified unrooted version of the tree with all the leaves removed and emphasizing just the existence of the groups mentioned above. To see the details concerning all the sequences, the same tree just in a circular version—based on the same alignment (Fig. S1)—has also been prepared as Figure S2.

The evolutionary tree thus shows the close relatedness between the trio of the main representatives of the *Thermotoga*-like group—the maltogenic amylases from *T. neapolitana*, *T. maritima* and *L. plantarum*—all of them occupying the adjacent branches (Fig. S2). The fourth characterized member of the subfamily, the maltogenic amylase from an uncultured bacterium, is located away from the remaining characterized representatives, surrounded mainly by putative proteins from bacteria of the Bacteroidota phylum. This group contains sequences of mainly bacterial members, but it also includes, albeit to a lesser extent, representatives of the Eukaryota (mainly the clade Protostomia—phyla Arthropoda, Rotifera and Mollusca, but also some algae, fungi and plants) and Archaea. It should be noted, that these do not form separate branches in the tree and are scattered among bacterial sequences. Concerning the second group, its main representative—the α-amylase from *H. japonica*—is positioned within the cluster grouping together its homologues from Haloarculaceae and the second characterized member, the α-amylase from *H. alexandrinus*. Interestingly, this group contains members of both Archaea and Bacteria that have not been mixed to each other. The bacterial group is, however, very small, consisting currently of only three members—one from the thermophilic and anaerobic bacterium *Caldithrix abyssi*, whereas the sources of the remaining two sequences are represented by unspecified bacteria (Fig. S2; Table S1).

**Fig. 2:**
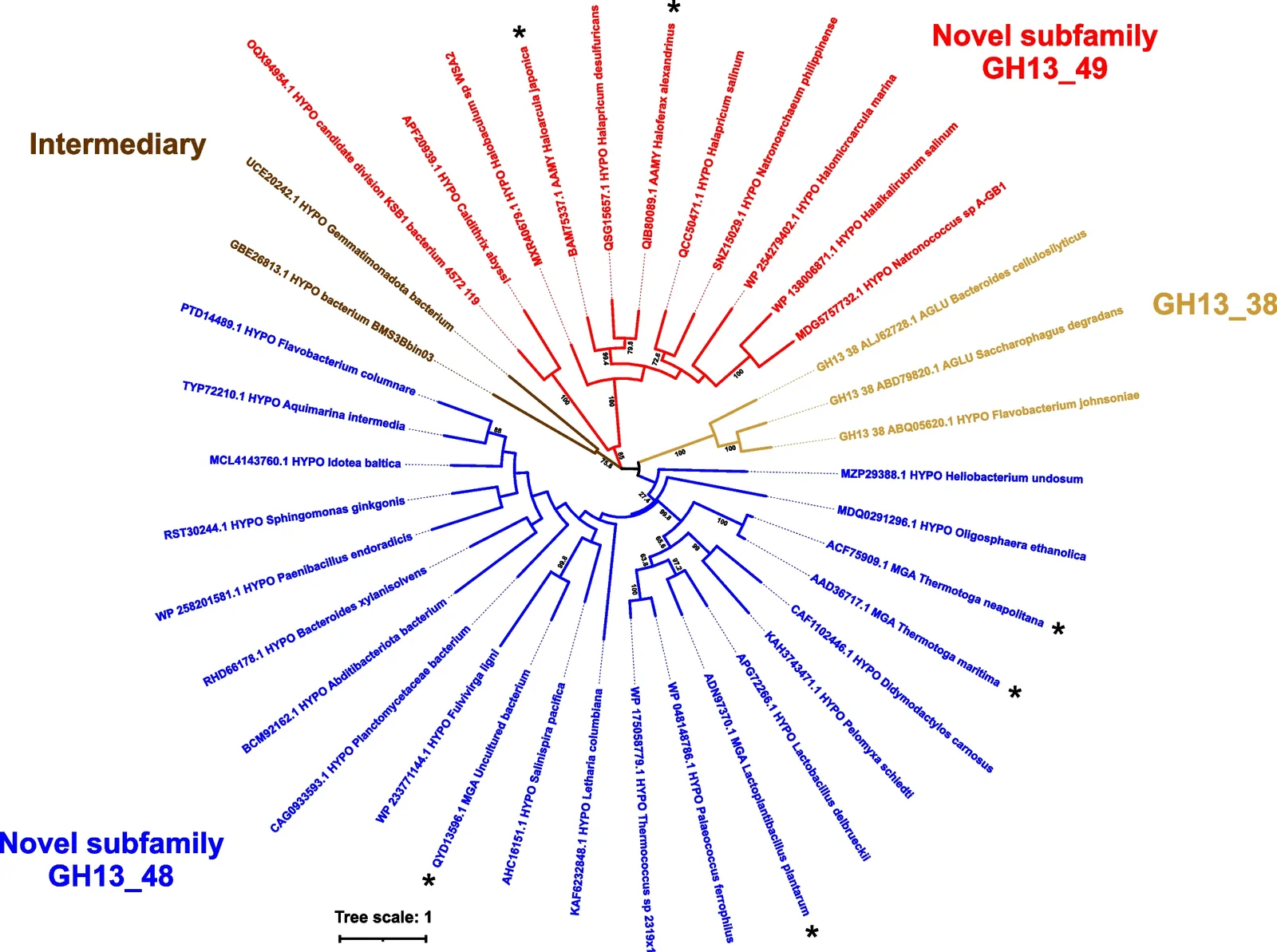
Evolutionary tree of a reduced sample of 38 selected sequences of interest. Sequence selection was performed to shed more light on the mutual relationships of the two new subfamilies GH13_48 and GH13_49 as well as on their relatedness to the intermediary group including the subfamily GH13_38. The tree is based on the alignment of full-length sequences (Fig. S3). Individual sequences are labelled with their GenBank accession numbers and the corresponding protein source; in the case of characterized enzymes, the accession number is followed by the protein abbreviation: AAMY α-amylase; MGA maltogenic amylase; AGLU α-glucosidase. The bootstrap values higher than 70% are shown

In order to investigate in a more detail the evolutionary relationships of the two groups, another evolutionary tree (Fig. 2) was calculated for just a reduced set of 38 selected full-length sequences of interest (Fig. S3). As it might be expected, the reduced evolutionary tree clearly demonstrates the existence of four independent groups represented by: (i) the *Thermotoga* group (around the maltogenic amylase from *T. neapolitana*); (ii) the *Haloarcula* group (around the α-amylase from *H. japonica*); (ii) the small group of two hypothetical proteins (currently without any specific classification); and (iv) the subfamily GH13_38 (Fig. 2). By inspecting the reduced tree in a detail, it seems that, within the *Thermotoga* group, the archaeal and eucaryotic members are integrated among their bacterial homologues more convincingly, while in the *Haloarcula* group, the two bacterial members appear to be more segregated from remaining archaeal representatives keeping their own separate branch (Fig. 2). These observations as well as the overall distribution of individual groups in the reduced evolutionary tree are supported by the relatively high bootstrap values (Fig. 2). In conclusion, the reduced evolutionary tree is in agreement with observations demonstrated by the overall evolutionary tree depicting the entire α-amylase family GH13 (Fig. 1).

Based on the above results, it appears that the two groups represented by the maltogenic amylase from *T. neapolitana* and the α-amylase from *H. japonica* form two novel distinct—although related—GH13 subfamilies. Hidden Markov models (Eddy 2011) generated for each subfamily in CAZy confirmed good discriminations between them, and therefore, from this point onwards in this article, and in the CAZy classification, the *Thermotoga*-like and *Haloarcula*-like groups will be designated, respectively, as subfamily GH13_48 and GH13_49.

### Specific sequence features of the two novel GH13 subfamilies

**Fig. 3:**
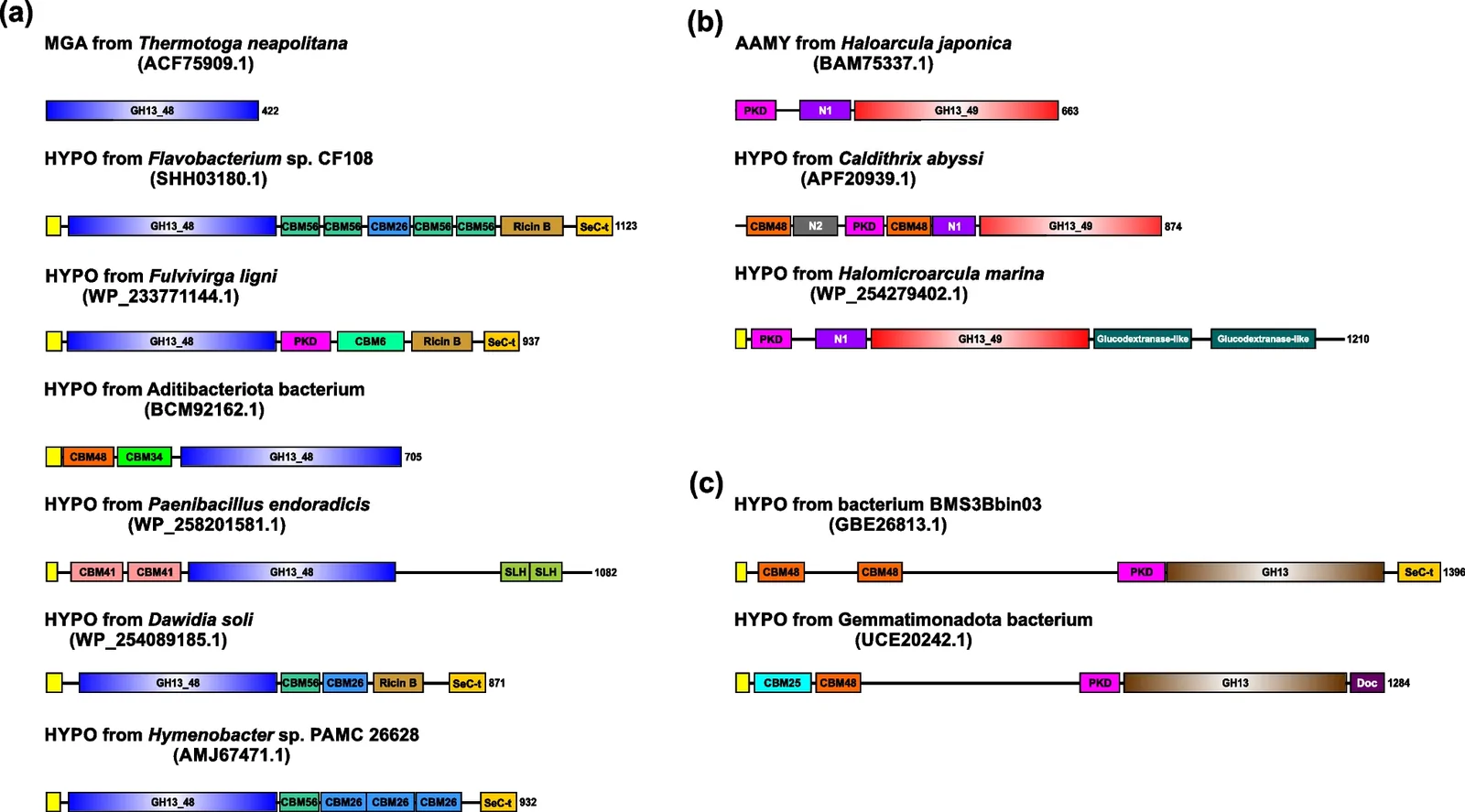
Domain arrangement of selected GH13 representatives used in the present study. Various domain compositions are illustrated in each of the three groups: **a** the novel subfamily GH13_48; **b** the novel subfamily GH13_49; and **c** the intermediary group. The individual domains are coloured as follows: the three GH13 canonical domains (including the catalytic TIM-barrel with inserted domain B and succeeding domain C)—blue/red/brown (depending on the group, which the enzyme or hypothetical protein belongs to); signal peptide—yellow; polycystic kidney disease (PKD) domain—magenta; N1—middle saturated purple; N2—grey; Ricin-B-like lectins (Ricin-B)—gold; secretion system C-terminal sorting domain (Sec-T)—tangerine yellow; glucodextranase-like—dark green; CBM6—navy blue; CBM25—cyan; CBM26—dodger blue; CBM34—green; CBM41—pink; CBM48—orange; CBM56—middle muted azure; surface layer homology (SLH)—middle muted chartreuse; dockerin—purple. The abbreviations MGA, AAMY and HYPO stand for the maltogenic amylase, α-amylase and hypothetical protein, respectively. The GenBank accession numbers of all selected enzymes and hypothetical proteins are given in parenthesis. The occurrences of individual members within the two newly established subfamilies are most frequently represented by their main representatives, i.e. the maltogenic amylase from *Thermotoga neapolitana*—277 cases of 304 sequences (more than 90%) and the α-amylase from *Haloarcula japonica*—28 cases of 41 sequences (almost 70%)

The domain arrangement of enzymes and hypothetical proteins from the set of 347 sequences brings additional arguments for the specificity of each group. In the GH13_48 subfamily, it comprises mainly just the three canonical GH13 domains, i.e. the catalytic TIM-barrel—domain A with inserted domain B and succeeded by the domain C. However, this three-domain basic arrangement can occasionally be accompanied at both ends by additional SBDs (CBM34, CBM48, CBM56 and CBM69), but also other domains (Fig. 3). Within the subfamily GH13_49, the domain architecture is more complex (Fig. 3). In most archaeal cases, the character of arrangement is as follows: polycystic kidney disease (PKD) domain, N1 domain and the GH13 A-B-C domains. Moreover, in some cases, the sequence also carries one or more copies of the so-called glucodextranase-like binding domain(s) (Fig. 3). Bacterial members of the GH13_49 are even more complex, two SBD copies of the family CBM48 being also involved (Fig. 3).

**Fig. 4:**
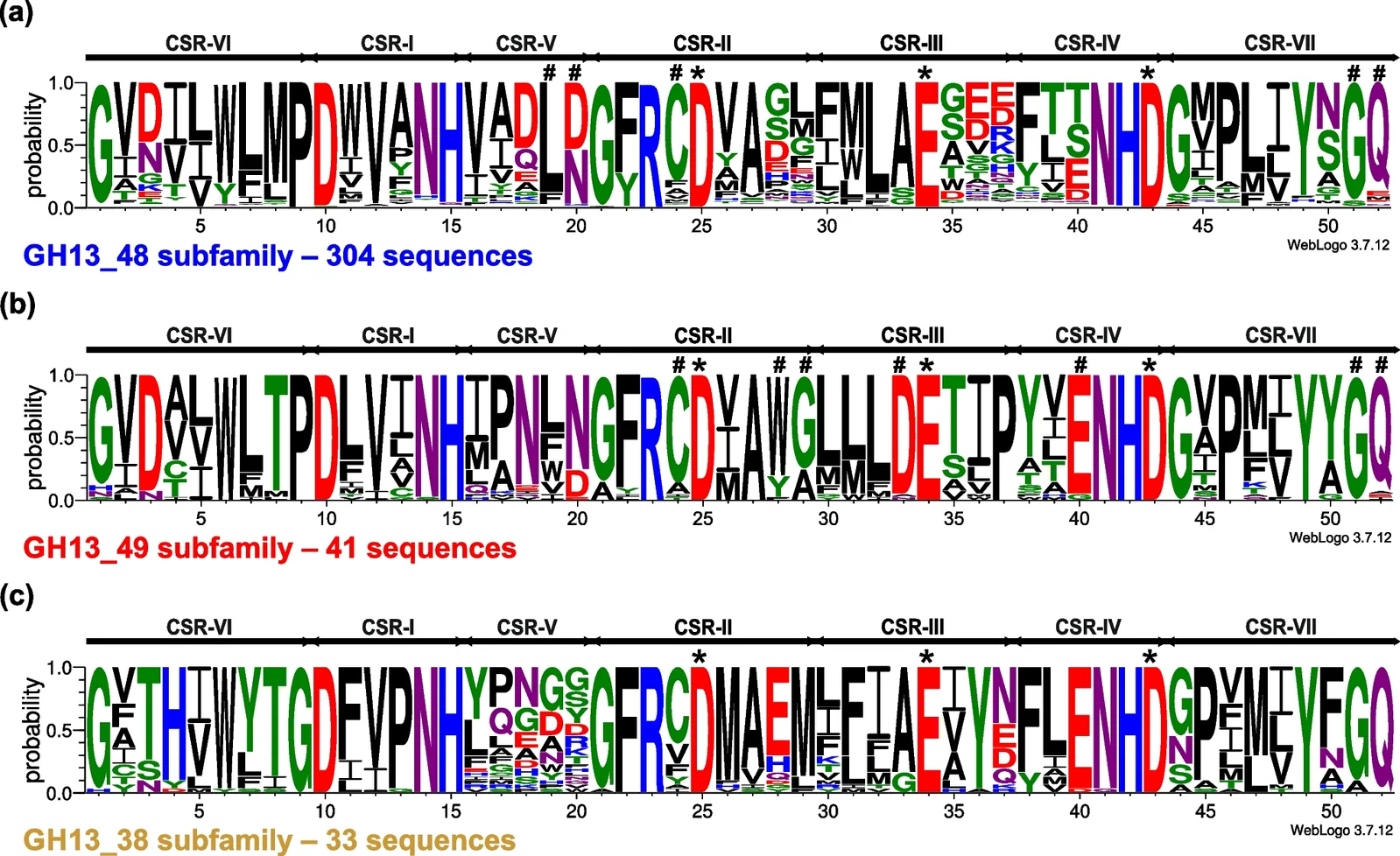
Sequence logos of the seven well-established GH13 CSRs of the two novel subfamilies: **a** the subfamily GH13_48 represented by the maltogenic amylase from *T. neapolitana* (blue; 304 sequences; representative sample of 6,325 sequences); **b** the subfamily GH13_49 formed around the α-amylase from *H. japonica* (red; 41 sequences; representative sample of 802 sequences). **c** For comparison, due to a close relatedness, the logo of the subfamily GH13_38 (colour, number of sequences) is also shown. CSR-I, residues 10–15; CSR-II, residues 21–29; CSR-III, residues 30–37; CSR-IV, residues 38–43; CSR-V, residues 16–20; CSR-VI, residues 1–9; CSR-VII, residues 44–52. The catalytic triad, i.e. the catalytic nucleophile (No. 25, aspartic acid), the proton donor (No. 34, glutamic acid) and the transition-state stabilizer (No. 43, aspartic acid) are indicated by asterisks. The well-conserved residues, which could represent specific features of the two novel GH13 subfamilies identified by the analysis, are indicated by a hashtag

A detailed inspection of amino acid sequences revealed several well-conserved positions across the 49 subfamilies within all seven CSRs (Fig. S1). For an easier comparison of stretches comprising the CSRs, sequence logos have been created for both novel subfamilies GH13_48 and GH13_49 (Fig. 4) as well as the subfamily GH13_38. The two novel subfamilies share some sequence features, but the logos contain also positions that distinguish them from each other. The highly conserved cysteine directly preceding the catalytic nucleophile in CSR-II (Fig. 4; position 24) seems to be just one of the most interesting shared sequence features. This cysteine is conserved in 81% of sequences if both groups are taken together; in remaining 19% being replaced mainly by alanine, valine, or leucine. The very well-conserved end of the CSR-VII as GQ (Fig. 4; position 51 and 52) may represent another sequence feature joining the two subfamilies (85.5% preservation). In the subfamily GH13_48, glycine may occasionally be substituted by serine or threonine, while glutamine may additionally be replaced mainly by methionine, aspartic, or glutamic acid. The glycine is invariably preserved in the GH13_49 subfamily, but glutamine can be exchanged for isoleucine, alanine, or glutamic acid in very few cases. In addition, these features, especially the end of the CSR-VII as well-conserved GQ can also be found in sequences of the members of the subfamily GH13_38 (Fig. 4) and also in members of a few other subfamilies—GH13_1; GH13_12; GH13_14 and GH13_40. The remaining common sequence features, such as the NH at the end of CSR-I (Fig. 4; positions 14 and 15), the GXR at the beginning of CSR-II (Fig. 4; positions 21–23), or also the NHD at the end of CSR-IV (Fig. 4; positions 41–43) are typical for most GH13 subfamilies (Janecek et al. 2014). It is, however, worth mentioning that, within the seven CSRs, these two groups bear also specific and highly conserved positions that distinguish them from each other, and even from representatives of already established GH13 subfamilies. Thus, for example, in the GH13_48 subfamily, the logo specifically exhibits two residues at the end of CSR-V well-conserved as L-[DN] (Fig. 4a; positions 19 and 20), conserved in 262 out of 304 sequences. In the GH13_49, highly specific positions are, e.g. the stretch at the end of CSR-II well-conserved as [WY]-[GA] (Fig. 4b; positions 28–29), a highly conserved aspartic acid just preceding the general acid/base in CSR-III (Fig. 4b; position 33), or well-preserved glutamic acid in the CSR-IV (Fig. 4b, position 40). The [WY]-[GA] segment is fully conserved in 40 of 41 sequences, while in the only remaining case, a third aromatic residue—phenylalanine, may alternate in the first position. Concerning the second feature, the aspartic acid just before the general acid/base (conserved in 39 out of 41 sequences) is substituted by alanine and asparagine. The preservation of the glutamic acid in the CSR-IV is also very high (39 out of 41 sequences) within the subfamily, with two cases of replacement by a glycine. Moreover, the latter feature is shared with representatives of the subfamily GH13_38 that also contain glutamic acid in that position (Fig. 4c, position 40), but in GH13_38 it is occasionally replaced by an aspartic acid. Note, the sequence logos are really meaningful, since the sequences used to create them represent the two large original groups of sequences—6325 for the GH13_48 and 802 for the GH13_49.

### Tertiary structure analysis

In an effort to reveal their closest structural homologues, the structures of the maltogenic amylases from *T. neapolitana* and *L. plantarum* (both the GH13_48) and the α-amylase from *H. japonica* (GH13_49) were superimposed with those of representatives of all 47 GH13 subfamilies established so far.

A complete overview of structure comparison is summarized in Table S2. The results have confirmed a close similarity (and thus also relatedness) between the maltogenic amylase from *T. neapolitana* and the *L. plantarum* enzyme supported by 283 corresponding C_α_ atoms with the root-mean square deviation (RMSD) of 1.04 Å. Furthermore, these comparisons have also demonstrated the closer relationships between the main representative of the novel subfamily GH13_48 with the GH13_38 α-glucosidase from *Bacteroides cellulosilyticus* (235 corresponding C_α_ atoms, RMSD 1.09 Å). Note, however, that no real structure is currently available for the GH13_38 subfamily; therefore, the AlphaFold-generated model was used. A close relatedness has also been confirmed for the two main representatives of the two novel subfamilies GH13_48 and GH13_49—228 corresponding C_α_ atoms with RMSD of 1.05 Å (Table S2). In addition, the GH13_45 α-amylase from *Geobacillus thermoleovorans* and the GH13_38 α-glucosidase from *B. cellulosilyticus* have also been identified as close homologues of GH13_49 α-amylase from *H. japonica* with characteristic values, i.e. 245 C_α_ atoms and RMSD of 1.09 Å for the former pair and 232 C_α_ atoms and RMSD of 1.02 Å for the latter pair of structures.

**Fig. 5:**
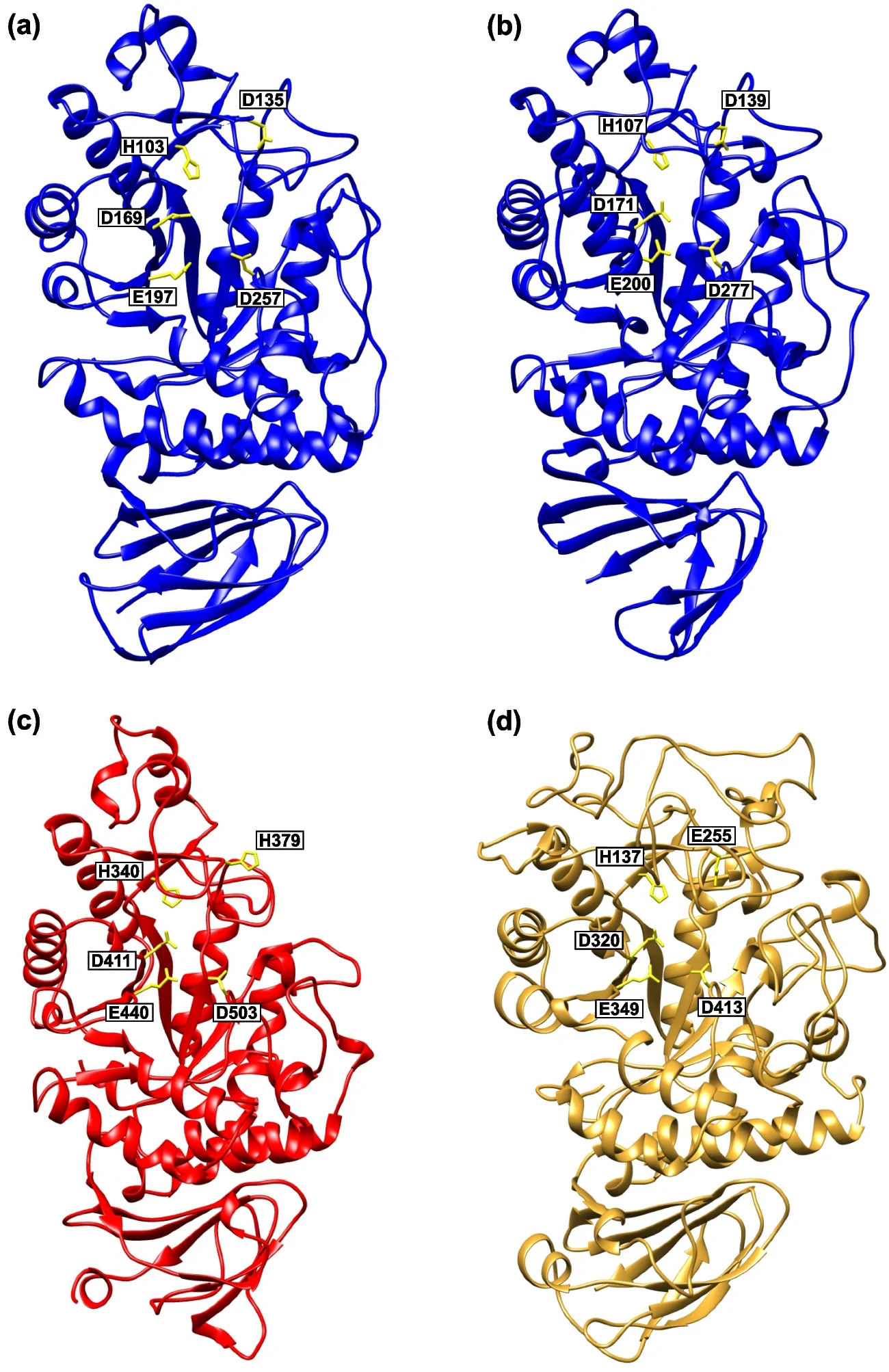
Tertiary structures (**a, b**) and AlphaFold-generated models (**c, d**) of the following representatives of the study: **a** maltogenic amylase from *T. neapolitana* (PDB: 4GKL; Jun et al. 2013); **b** maltogenic amylase from *L. plantarum* (PDB: 3DHU; unpublished); **c** α-amylase from *H. japonica* (UniProt: L8B068); **d** α-glucosidase from *B. cellulosilyticus* (UniProt: A0A0P0GJC2). Catalytic residues are displayed as side chains and coloured yellow. The residues that are involved in the non-reducing end carbohydrate-binding site of the maltogenic amylase from *T. neapolitana*—Asp135 and His103 and corresponding residues in other structures/models—are also shown

The structure gallery of selected examples (Fig. 5) emphasizes not only the overall similarity of GH13 members studied here, but also shows the side chains of their catalytic machinery. Furthermore, the residues involved in the so-called non-reducing end carbohydrate-binding site of the GH13_48 maltogenic amylase from *T. neapolitana*, the Asp135 and His103 (Jun et al. 2013), are also displayed (Fig. 5). The His103 located in CSR-I belongs to a highly conserved position throughout the representatives of the entire family GH13 (Fig. 4; position 15; Fig. 5; Fig. S1). Interestingly, the former one, i.e. the aspartic acid residue has been found conserved in the maltogenic amylase from *L. plantarum* and also in 95.4% sequences (290 out of 304) of GH13_48 members of the studied dataset (Fig. S1). On the other hand, it is not conserved in other subfamilies or groups of enzymes and putative proteins. With regard to GH13_38 α-glucosidase from *B. cellulosilyticus*, that aspartic acid has been observed to be substituted by a glutamic acid (Fig. 5).

## Discussion

The main representatives of the present *in silico* study include amylolytic enzymes from the α-amylase family GH13 that were characterized and/or their three-dimensional structures were solved several years ago. The novel subfamily GH13_48 is represented by five enzymes—the maltogenic amylase from *T. neapolitana*, its counterpart from *T. maritima*, metagenomic-derived enzyme referred to as PersiAmy1 and two enzymes with identical sequences from two different strains of *L. plantarum*—WCFS1 and ST-III. Among them, the first one is the best-studied enzyme, since it has been characterized as maltogenic amylase (Park et al. 2010) and its tertiary structure has also been determined (Jun et al. 2013). Although its counterpart from *T. maritima* has been cloned and characterized as an α-amylase (Lim et al. 2003), no end-product analysis was performed. A similar case is represented by the enzyme PersiAmy1, which was identified through rumen metagenome screening (Ariaeenejad et al. 2021). This enzyme, being also designated just as an α-amylase without its product profile analysis, is capable of hydrolyzing the α-1,6-glycosidic linkages in pullulan (Ariaeenejad et al. 2021). The last two sequence identical enzymes from two strains of *L. plantarum* were biochemically characterized as maltogenic amylases. In the case of the strain ST-III, reaction pattern analysis revealed hydrolysis of 4-nitrophenyl-α-D-maltopentaoside from the non-reducing end (Plaza-Vinuesa et al. 2019), whereas the enzyme of the WCFS1 strain hydrolysed maltopentaose and dextrins only (Jeon et al. 2016). Both studies thus confirmed the exo-type action of hydrolysis of the α-glucosidic bonds (Jeon et al. 2016; Plaza-Vinuesa et al. 2019). Moreover, for the maltogenic amylase from *L. plantarum* WCFS1 strain, the three-dimensional structure coordinates have been deposited in PDB (PDB code: 3DHU), but still without associated publication. Concerning the subfamily GH13_49, the main characterized member, the α-amylase from extremely halophilic archaeon *H. japonica* exhibits activity toward soluble starch, amylose, amylopectin and glycogen (Onodera et al. 2013). The second characterized member is the α-amylase from *H. alexandrinus*, the enzymatic activity of which was tested using starch agar plate assay (Verma et al. 2020). To shed some light on the evolutionary position of the seven enzymes mentioned above in the overall context of the α-amylase family GH13, related hypothetical proteins have been obtained, and subsequently a detailed bioinformatics analysis has been performed.

In an attempt to indicate the positions of the two novel subfamilies GH13_48 and GH13_49 in the entire α-amylase family GH13 context, a maximum-likelihood evolutionary tree was constructed (Fig. 1; a detailed circular version of the same tree with all the leaves described is shown in Figure S2). The tree (based on the alignment shown in Figure S1) includes, in addition to the studied set of 347 sequences, three sequences for each of the 47 GH13 subfamilies established so far (Table S1). The evolutionary tree reflects several well-known relationships recognized among the GH13 subfamilies previously, e.g.: (i) clustering the liquefying α-amylases from bacteria, plants and hyperthermophilic archaeons classified in subfamilies GH13_5, GH13_6 and GH13_7, respectively (Janecek et al. 1999; Jones et al. 1999; van der Kaaij et al. 2007; Mieog et al. 2017); (ii) grouping together the α-amylases from actinomycetes and animals of subfamilies GH13_32, GH13_15 and GH13_24, respectively (Janecek 1994; Pujadas and Palau 2001; Da Lage et al. 2004, 2007; Janickova and Janecek 2020); and (iii) a common evolutionary history of oligo-1,6-glucosidase and neopullulanase groups (Oslancova and Janecek 2002) with heavy chains of amino acid transporters 4F2 antigen and rBAT protein from subfamilies GH13_34 and GH13_35, respectively (Janecek et al. 1997; Gabrisko and Janecek 2009; Janecek and Gabrisko 2016; Fort et al. 2021). Interestingly, a similar evolutionary relatedness has been observed between the two novel subfamilies GH13_48 and GH13_49, whose representatives are located in two clusters adjacent to each other in the phylogenetic tree (Fig. 1). These subfamilies are on a long branch distant from the remaining GH13 subfamilies, except for the GH13_38. Thus, of all 47 currently established GH13 subfamilies, the subfamily GH13_38 appears to be the only one that is closely related to both GH13_48 and GH13_49. Based on experimental characterization of the enzymes from *Bacteroides cellulosilyticus* and *Saccharophagus degradans*, the subfamily GH13_38 was assigned the α-glucosidase specificity (Helbert et al. 2019). There is also a small cluster of just two hypothetical proteins located in the same part of the tree, which has been labelled as an intermediary group (Fig. 1). Although these sequences seem to may eventually form a base for the future GH13 subfamily, without at least a single characterized representative—according to the policy in the CAZy classification—it is currently not actual to create a new subfamily from this intermediary group.

Further, to provide a more detailed view of the part of the tree containing the two new subfamilies, a reduced evolutionary tree (based on the full-length alignment of 38 selected sequences; Table S1) was also calculated (Fig. 2). In fact, distribution of sequences to four separate clusters and all other findings observed in both trees are in agreement with each other. Indeed, GH13_38 α-glucosidases appear to be most closely related group to the two novel subfamilies GH13_48 and GH13_49, as suggested also by some shared sequence features. However, as the tree with the reduced number of 38 sequences shows, due to the low bootstrap values obtained, this observation should still be taken into consideration with some limitation (Fig. 2). Another feature of interest seen in novel GH13 subfamilies may be represented by the integration of the archaeal and eukaryotic representatives into the bacterial counterparts in the subfamily GH13_48, whereas on the other hand, in subfamily GH13_49, the two bacterial representatives are strictly separated on their own branch adjacent to all archaeal members of this subfamily. These findings are also accompanied by the higher bootstrap values of the given branches (Fig. 2). In principle, the horizontal gene transfer could be considered mainly in the subfamily GH13_48 since its eukaryotic members are not found throughout this domain (Table S1). Moreover, the horizontal gene transfer was observed also in other GH13 subfamilies previously (Da Lage et al. 2004, 2013; Da Lage 2018; Desiderato et al. 2020).

In spite of their overall similarities, the representatives of the two new GH13 subfamilies possess a domain architecture that emphasizes also their differences. As mentioned above, most representatives of the GH13_48 subfamily consist only of the three GH13 canonical domains, i.e. the A + B + C domains. In some cases, however, members of this subfamily form, with the presence of additional domains, more complex structures. Therefore, SBDs (CBM26, CBM34, CBM41, CBM48 and CBM69), other CBMs (CBM6 and CBM56), or even further domains (e.g. SLH; dockerin; Ricin-B-like) may be attached at both ends of their protein molecules (Fig. 3). The SBDs are well recognized as auxiliary domains of a relatively large number of the amylolytic enzymes from the GH13 family (Janecek et al. 2019). The CBM domains, in general, do not display enzymatic activity but are involved in the targeting of the catalytic domain to the saccharide substrates to process it in the active site (Boraston et al. 2004). These domains from other families are responsible for adhesion to other types of carbohydrates, e.g. CBM6 binds cellulose (Fernandes et al. 1999), while CBM56 has a more general β-1,3-glucan binding function (Yamamoto et al. 1998). However, the functionality of these non-SBD CBMs in the family GH13 enzymes remains still unclear. The length of the sequences of the subfamily GH13_49 members is longer and even more complex. Typically, there are two main types of domain architecture—archaeal and bacterial ones (Fig. 3). In both cases, the additional domains are involved—CBM48, PKD and other domains of unknown function (N1 and N2) (Fig. 3). The PKD domain is a module originally found in an extracellular segment of the large cell-surface glycoprotein polycystin-1 (Hughes et al. 1995). Although the function of this domain is still poorly understood, due to its known tertiary structure, it could be involved in protein–protein and protein-carbohydrate interactions (Bycroft et al. 1999). Importantly, both new subfamilies as well as the two intermediary putative proteins share domains like CBM48 or PKD, underscoring their obvious relatedness (Fig. 3).

Since the primary structure analysis was focused mainly on seven well-established CSRs (Janecek 2002), sequence logos were generated of these CSRs for both of the two novel subfamilies and for the GH13_38 (Fig. 4). It should be noted that within the GH13 family, some subfamilies share certain sequence features within their CSRs, supporting thus their closely evolutionary relatedness, e.g. the so-called oligo-1,6-glucosidase group and the neopullulanase group (Oslancova and Janecek 2002). On the other hand, it is simultaneously possible—based on just the presence of some unique features in CSRs—to distinguish representatives of individual GH13 subfamilies from each other. This can also be the case for the two above-mentioned groups since the unique sequence in CSR-V is QpDln and MpKln for members of the oligo-1,6-glucosidase and the neopullulanase group, respectively (Oslancova and Janecek 2002; Majzlova et al. 2013). Furthermore, representatives of the subfamily GH13_45, whether they possess the classical or aberrant catalytic triad, contain the motif LPDlx in the CSR-V as their typical feature (Puspasari et al. 2013; Janecek et al. 2015; Sarian et al. 2017). As a recent example, the members of the subfamily GH13_46 can be considered with a characteristic aromatic end of the CSR-II and a well-conserved glutamic acid directly succeeding the proton donor in CSR-III (Marecek and Janecek 2022).

It is thus not surprising at all that the two novel subfamilies GH13_48 and GH13_49 delivered in the present study also share some sequence characteristics, but at the same time, they can be distinguished from each other by some other unique features. The highly conserved cysteine directly preceding the catalytic nucleophile in CSR-II (Fig. 4) may be just one of the most significant features common to both new subfamilies. Another one could be represented by the well-conserved end of the CSR-VII as GQ (Fig. 4). It is worth mentioning here that these two features together may simultaneously distinguish GH13_48 and GH13_49 from all remaining GH13 subfamilies established so far. The GQ feature at the end of the CSR-VII can also be observed in GH13_38 members (Fig. 4c) as well as in subfamilies GH13_1, GH13_12, GH13_14 and GH13_40. In general, however, both new subfamilies do contain sequence features that have been typically recognized in most GH13 subfamilies previously (MacGregor and Svensson 1989; Jespersen et al. 1991, 1993; Janecek et al. 1999, 2015; Janecek 2002; Oslancova and Janecek 2002; Puspasari et al. 2013; Majzlova et al. 2013; Janecek and Zamocka 2020), such as: (i) NH at the end of CSR-I (Fig. 4; positions 14 and 15); (ii) GXR at the beginning of CSR-II (Fig. 4; positions 21–23); and (iii) NHD at the end of CSR-IV (Fig. 4; positions 41–43). In addition, the subfamily GH13_48, represented by the maltogenic amylase from *T. neapolitana*, differs in particular in the sequence at the end of the CSR-V, which is well-conserved as L-[DN] (Fig. 4a; positions 19 and 20). The second subfamily GH13_49, formed around the α-amylase from *H. japonica*, can be distinguished by the stretch of residues at the end of CSR-II that is well-conserved as [WY]-[GA] (Fig. 4b; positions 28–29), highly conserved aspartic acid just preceding the general acid/base (Fig. 4b; position 33), or by preservation of the glutamic acid within the CSR-IV (Fig. 4b; position 40). The last mentioned feature, a well-conserved glutamic acid in the CSR-IV, is also characteristic for members of the subfamily GH13_38 (Fig. 4c). Remarkably, during the completing the sequence set, some bacterial representatives were identified in the GH13_48 subfamily, which did not contain one or more catalytic residues. Although these proteins may lack the enzymatic activity, it is worth mentioning here that in the subfamily GH13_45 the protein BmaN1 from *Bacillus megaterium* with an aberrant catalytic triad has already been biochemically characterized as an active amylolytic enzyme (Sarian et al. 2017).

Finally, in order to identify the closest structural homologues for selected representatives of the two new GH13 subfamilies delivered in the present study, their real three-dimensional structures or AlphaFold-generated models were superimposed with those of representatives of all 47 existing GH13 subfamilies (Table S2). The spatial structure comparison has indicated the closest homology of representatives of GH13_48 to GH13_38. It is necessary to point out, however, that no real tertiary structure has been determined in the subfamily GH13_38, so only the *B. cellulosilyticus* α-glucosidase AlphaFold-generated model was used for comparison. Some clear sequence-structural differences between GH13_38 and GH13_48 have also been detected, mainly the difference in the length of domain B, which is the extended loop of variable length and sequence protruding out from the catalytic TIM-barrel between the strand β3 and helix α3 (Janecek et al. 1997; MacGregor et al. 2001). Thus, while the domain B in the subfamily GH13_38 consists of ~ 80–90 residues, in the novel subfamily GH13_48, it could be only ~ 30–40 residues long. The superposition results have also confirmed the close relatedness of the two novel subfamilies GH13_48 and GH13_49 (Table S1). Especially, within the catalytic domain, both subfamilies resemble each other very well (Fig. 5). However, this is not the case with regard to overall domain composition. While the GH13_48 subfamily representatives usually do not contain any domains additional to GH13 canonical three-domain arrangement (MacGregor et al. 2001; Janecek et al. 2014), in the subfamily GH13_49, the PKD and N1 domains precede the catalytic TIM-barrel (Fig. 3). Beside the GH13_38 α-glucosidases, the members of the subfamily GH13_45 seem to be the further structural homologues close to GH13_49. It is of note that despite the original proposal to establish this subfamily almost 10 years ago (Janecek et al. 2015), the GH13_45 was created relatively recently including also the enzymes with the so-called aberrant catalytic triad (Sarian et al. 2017).

A very close structural similarity has also been confirmed for the maltogenic amylases from *T. neapolitana* and *L. plantarum* (Table S1). This could be expected since they both come from the same subfamily GH13_48. The attention was further paid to Asp135 and His103 of the maltogenic amylase from *T. neapolitana* demonstrated to be involved in the non-reducing end carbohydrate-binding site and located on top of the active-site cleft of the enzyme (Jun et al. 2013). The histidine residue is located in the CSR-I (Fig. 4; position 15) and belongs to one of most highly conserved positions throughout the whole α-amylase family GH13 (Janecek et al. 2014). The mentioned aspartic acid positioned just before the CSR-V is highly conserved (95.4%) in the subfamily GH13_48, while in other subfamilies it is not, indicating the possible unique role, in the subfamily GH13_48, also for the His103. Mutational analyses of the Asp135 revealed its importance for substrate recognition, but not in a direct involvement in the catalytic mechanism (Jun et al. 2013). The residue Asp135 of the maltogenic amylase from *T. neapolitana* is therefore likely to play an important role in substrate recognition throughout the subfamily. It is thus reasonable to assume that all GH13_48 members might act on the substrate in a similar mode of action, i.e. to possess the maltogenic amylase enzyme specificity.

In summary, it can be concluded that the two newly proposed GH13 groups described in the present study deserve to define the new subfamilies—GH13_48 and GH13_49. They represent two closely related but still independent groups in the overall context of the α-amylase family GH13. Although they contain some sequence features that they share, at the same time they carry other sequence features that discriminate them from each other.

## Supplementary Information

Below is the link to the electronic supplementary material.

Supplementary file1 (PDF 7.17 MB)

## Data Availability

The sequence datasets generated and/or analysed during the current study are available in the Supplementary material.
