# Supplementary material for: Two newly established and mutually related subfamilies GH13_48 and GH13_49 of the α-amylase family GH13
Source: Appl Microbiol Biotechnol. 2024 Jul 11;108(1):415. doi: 10.1007/s00253-024-13251-x (PMC11239784; doi:10.1007/s00253-024-13251-x)
Supplement: Supplementary file 1 — Supplementary file1 (PDF 7.17 MB) [file 253_2024_13251_MOESM1_ESM.pdf]

Supplementary material

Journal name:
Applied Microbiology and Biotechnology

Manuscript Title:
Two newly established and mutually related subfamilies GH13_48 and GH13_49 of the α-amylase family GH13

The name(s) of the author(s):
Filip Mareček[1]*, Nicolas Terrapon[2] & Štefan Janeček[1,3]*

The affiliation(s) and address(es) of the author(s):
[1] *Laboratory of Protein Evolution, Institute of Molecular Biology, Slovak Academy of Sciences, SK-84551 Bratislava, Slovakia*
[2] *Architecture et Fonction des Macromolécules Biologiques, UMR CNRS, Aix-Marseille University,USC INRAE, F-13288 Marseille, France*
[3] *Department of Biology, Institute of Biology and Biotechnology, Faculty of Natural Sciences, University of SS. Cyril and Methodius, SK-91701 Trnava, Slovakia*

The e-mail address, telephone and fax numbers of the corresponding author:
e-mail: *Stefan.Janecek@savba.sk*; telephone: ++421-2-59307420; fax: ++421-2-59307416

Table S1. List of sequences used in the present study.[a]

No.	Kingdom	Protein[b]	Subfamily[c]	Source	GenBank	UniProt	Length
1.	Bacteria	HYPO	GH13_48	Flavobacteriales bacterium	RZP12244.1	UPI00120AD95E	458
2.	Bacteria	HYPO	GH13_48	Flavobacteriales bacterium	RZP08053.1	A0A520UMA6	443
3.	Bacteria	HYPO	GH13_48	Flavobacteriales bacterium CG_4_9_14_3_um_filter_40_17	PJB12981.1	A0A2M8A6N6	457
4.	Bacteria	HYPO	GH13_48	*Imtechella halotolerans* K1	EID76795.1	I0WK79	497
5.	Bacteria	HYPO	GH13_48	*Kordia algicida* OT-1	EDP96606.1	A9DQ94	465
6.	Bacteria	HYPO	GH13_48	Uncultured *Dokdonia* sp.	WP_298329650.1	UPI00262331C0	480
7.	Bacteria	HYPO	GH13_48	*Aquimarina intermedia*	TYP72210.1	A0A5S5C1J5	460
8.	Eucarya	HYPO	GH13_48	*Potamilus streckersoni*	KAK3604785.1	-	830
9.	Bacteria	HYPO	GH13_48	*Pseudomaricurvus alkylphenolicus*	WP_166989327.1	UPI0014235C69	463
10.	Bacteria	HYPO	GH13_48	Bacteroidia bacterium	PID89777.1	A0A2G6GT70	470
11.	Bacteria	HYPO	GH13_48	Bacteroidales bacterium	UCH15058.1	UPI001EA6CE92	498
12.	Bacteria	HYPO	GH13_48	Bacteroidota bacterium	RLD19591.1	A0A661XIZ2	498
13.	Bacteria	HYPO	GH13_48	Ignavibacteria bacterium ADurb.Bin266	OQA66704.1	A0A1V5TJ53	440
14.	Bacteria	HYPO	GH13_48	*Melioribacter roseus* JCM 17771	AFN75430.1	I6Z8F1	454
15.	Bacteria	HYPO	GH13_48	*Ornithobacterium rhinotracheale*	QAR31169.1	A0A410JT36	474
16.	Bacteria	HYPO	GH13_48	*Profundicola chukchiensis*	MDG4951057.1	UPI00243E898E	464
17.	Bacteria	HYPO	GH13_48	Flavobacteriaceae bacterium UJ101	APD06119.1	A0A1J0LKH0	473
18.	Bacteria	HYPO	GH13_48	Uncultured *Paludibacter* sp.	VBB45750.1	A0A653ACG2	466
19.	Bacteria	HYPO	GH13_48	Crocinitomicaceae bacterium	GIR57489.1	A0A920KC24	443
20.	Bacteria	HYPO	GH13_48	*Fluviicola* sp. XM-24bin1	PWL31337.1	A0A316KAL1	445
21.	Bacteria	HYPO	GH13_48	*Longitalea* sp. SCSIO 12813	WP_306640064.1	UPI0027BAF1AE	467
22.	Bacteria	HYPO	GH13_48	Bacteroidota bacterium	TNE78542.1	A0A9E6W164	436
23.	Bacteria	HYPO	GH13_48	*Fulvitalea axinellae*	BDD08929.1	-	475
24.	Bacteria	HYPO	GH13_48	*Odoribacte rlaneus* CAG:561	CCZ81349.1	R5UZV4	456
25.	Bacteria	HYPO	GH13_48	Bacteroidota bacterium	PCJ63739.1	A0A2A5E5N2	466
26.	Bacteria	HYPO	GH13_48	*Croceimicrobium hydrocarbonivorans*	QNR25628.1	A0A7H0VIT0	456
27.	Bacteria	HYPO	GH13_48	*Hyphobacterium* sp. CCMP332	QNL22394.1	UPI001650C71D	457
28.	Bacteria	HYPO	GH13_48	Bacteroidetes bacterium GWF2_33_16	OFX89561.1	A0A1F3ILE2	455
29.	Bacteria	HYPO	GH13_48	Bacteroidota bacterium	RCL63763.1	A0A368CX14	466
30.	Bacteria	HYPO	GH13_48	*Roseivirga pacifica*	WP_252938339.1	UPI002094CABC	456
31.	Bacteria	HYPO	GH13_48	*Litoribacter populi*	WP_143960756.1	UPI0011803072	457
32.	Bacteria	HYPO	GH13_48	*Aliifodinibius salipaludis*	WP_141239846.1	UPI001140C371	465
33.	Bacteria	HYPO	GH13_48	*Coraliomargarita* sp. SDUM461003	MDQ8208009.1	-	494
34.	Bacteria	HYPO	GH13_48	Balneolaceae bacterium	TVR31165.1	A0A6I7QD74	463
35.	Bacteria	HYPO	GH13_48	*Cyclonatronum* sp.	MCH8485807.1	-	455
36.	Bacteria	HYPO	GH13_48	Ignavibacteria bacterium CG1_02_37_35	OIO17781.1	A0A1J4U4P4	446
37.	Bacteria	HYPO	GH13_48	Calditrichota bacterium	KAA3611096.1	UPI00139417D9	456
38.	Bacteria	HYPO	GH13_48	Ignavibacteriales bacterium	QOJ29674.1	A0A7S6SFP5	456
39.	Bacteria	HYPO	GH13_48	Bacteroidia bacterium	PIE86166.1	A0A2G6PNK8	425
40.	Bacteria	HYPO	GH13_48	Bacteroidota bacterium	KAF0200891.1	A0A7J5VYG6	429
41.	Bacteria	HYPO	GH13_48	Bacteroidetes bacterium CG2_30_33_31	OIP00198.1	A0A1J5B008	457

42.	Bacteria	HYPO	GH13_48	Rhodothermaeota bacterium MED-G12	PDH57537.1	A0A2A5Y4V2	464
43.	Bacteria	HYPO	GH13_48	Crocinitomicaceae bacterium TMED209	RPG87855.1	A0A424R8P7	498
44.	Bacteria	HYPO	GH13_48	Bacteroidota bacterium	TNE50071.1	-	473
45.	Eucarya	HYPO	GH13_48	*Darwinula stevensoni*	CAD7252417.1	A0A7R9AE57	1593
46.	Bacteria	HYPO	GH13_48	*Fibrella aestuarina* BUZ 2	CCH01189.1	I0KAN6	476
47.	Bacteria	HYPO	GH13_48	*Vibrio sinaloensis* DSM 21326	EGA68405.1	E8MCA0	411
48.	Bacteria	HYPO	GH13_48	*Vibrio* sp. JCM 19052	GAK19705.1	A0A061PY55	366
49.	Bacteria	HYPO	GH13_48	Planctomycetes bacterium Pla133	QDU68335.1	A0A518BMY4	416
50.	Bacteria	HYPO	GH13_48	*Hymenobacter psoromatis*	AMR27413.1	A0A142HEV5	446
51.	Bacteria	HYPO	GH13_48	Calditrichota bacterium	RMH64054.1	A0A3M2FT05	451
52.	Bacteria	HYPO	GH13_48	*Undibacterium flavidum*	MBC3875504.1	UPI00164B20D4	451
53.	Bacteria	HYPO	GH13_48	Muribaculaceae bacterium	GFI12946.1	A0A7J0AGG4	467
54.	Bacteria	HYPO	GH13_48	*Prevotella* sp. CAG:485	CDE06671.1	R7EW74	483
55.	Bacteria	HYPO	GH13_48	Bacteroidota bacterium	RLD43041.1	A0A497CUD0	464
56.	Bacteria	HYPO	GH13_48	*Duganella levis*	MYN29304.1	UPI001369E1BF	430
57.	Bacteria	HYPO	GH13_48	*Sphingomonas ginkgonis*	RST30244.1	A0A3R9X6W2	435
58.	Eucarya	HYPO	GH13_48	*Ricinus communis*	EEF24336.1	B9TIH8	371
59.	Bacteria	HYPO	GH13_48	*Novosphingobium* sp. NDB2Meth1	WP_072382030.1	UPI000931E079	439
60.	Bacteria	HYPO	GH13_48	*Roseateles puraquae*	OWR02431.1	A0A254N2N8	409
61.	Bacteria	HYPO	GH13_48	*Breznakibacter xylanolyticus*	PZX20730.1	A0A2W7NLH0	459
62.	Bacteria	HYPO	GH13_48	Bacteroidetes bacterium ADurb.Bin041	OQC35924.1	A0A1V6HCV2	456
63.	Bacteria	HYPO	GH13_48	*Geofilum rhodophaeum*	WP_088653137.1	UPI000B528A93	455
64.	Bacteria	HYPO	GH13_48	*Alistipes* sp. CAG:268	CDC99258.1	R6VL17	446
65.	Bacteria	HYPO	GH13_48	Bacteroidetes bacterium ADurb.Bin139	OQB69698.1	A0A1V6BYK8	436
66.	Bacteria	HYPO	GH13_48	Bacteroidales bacterium	MBR0110455.1	-	996
67.	Bacteria	HYPO	GH13_48	Bacteroidia bacterium	GHU94128.1	UPI00221B81C9	413
68.	Bacteria	HYPO	GH13_48	*Millionella massiliensis*	WP_274957478.1	-	443
69.	Bacteria	HYPO	GH13_48	*Acetobacteroides hydrogenigenes*	TCN64708.1	A0A4R2E807	417
70.	Bacteria	HYPO	GH13_48	Uncultured *Alistipes* sp.	WP_295936620.1	-	425
71.	Bacteria	HYPO	GH13_48	*Alistipes* sp. CAG:831	CCY36953.1	R5I4E6	442
72.	Bacteria	HYPO	GH13_48	*Proteiniphilum* sp. UBA4988	WP_238867769.1	UPI001EEBAC10	451
73.	Bacteria	HYPO	GH13_48	Bacteroidetes bacterium 41-46	OJV18238.1	A0A1M3CZ83	449
74.	Bacteria	HYPO	GH13_48	*Tenuifilum* sp.	MDI3527941.1	UPI0024AB47BA	458
75.	Bacteria	HYPO	GH13_48	*Phaeocystidibacter luteus*	KAB2813838.1	A0A6N6RHI1	472
76.	Bacteria	HYPO	GH13_48	Cryomorphaceae bacterium	QTN38589.1	A0A975IA06	475
77.	Bacteria	HYPO	GH13_48	Bacteroidota bacterium	PTL98760.1	A0A2T4WB44	484
78.	Bacteria	HYPO	GH13_48	*Marivirga lumbricoides*	GGC45001.1	UPI001669BDA9	458
79.	Bacteria	HYPO	GH13_48	Cryomorphaceae bacterium BACL7 MAG-120910-bin2	KRO58761.1	A0A0R2R802	459
80.	Bacteria	HYPO	GH13_48	Saprospirales bacterium	TVQ46139.1	A0A6N2CV23	450
81.	Eucarya	HYPO	GH13_48	*Idotea baltica*	MCL4143760.1	UPI0026E707C1	391
82.	Bacteria	HYPO	GH13_48	Sphingobacteriales bacterium BACL12 MAG-120802-bin5	KRP09954.1	A0A0R2VDQ3	443
83.	Bacteria	HYPO	GH13_48	*Hydrotalea sandarakina*	PZX61841.1	A0A2W7RNH4	454
84.	Bacteria	HYPO	GH13_48	*Paraflavitalea devenefica*	NII25694.1	UPI0014216E67	460
85.	Bacteria	HYPO	GH13_48	Sphingobacteriia bacterium	TAF56997.1	A0A976H517	449
86.	Bacteria	HYPO	GH13_48	Chitinophagaceae bacterium	RYY66426.1	A0A4Q5USN6	478
87.	Bacteria	HYPO	GH13_48	*Roseivirga pacifica*	QCK16385.1	A0A4D7K0A2	459

88.	Bacteria	HYPO	GH13_48	*Aestuariivivens sediminicola*	WP_242121039.1	UPI001F5ADFE6	456
89.	Bacteria	HYPO	GH13_48	*Fodinibius halophilus*	NGP87033.1	A0A6M1T338	464
90.	Bacteria	HYPO	GH13_48	*Fischerella thermalis* CCMEE 5319	PMB22228.1	UPI000CAFF25D	461
91.	Bacteria	HYPO	GH13_48	*Agathobacter ruminis*	PHU37968.1	A0A2G3E3W7	476
92.	Bacteria	HYPO	GH13_48	*Butyrivibrio* sp. MC2013	WP_196803062.1	UPI0018C9D5DB	519
93.	Bacteria	HYPO	GH13_48	*Butyrivibrio fibrisolvens* AB2020	WP_022758533.1	UPI0003B4411D	462
94.	Bacteria	HYPO	GH13_48	*Roseburia* sp. MSJ-14	WP_216555785.1	UPI001C100B99	495
95.	Bacteria	HYPO	GH13_48	*Eubacterium* sp. CAG:603	CCZ03538.1	R5NIH8	470
96.	Bacteria	HYPO	GH13_48	*Paenibacillus wynnii*	WP_052087767.1	UPI00068F4FA2	1100
97.	Bacteria	HYPO	GH13_48	*Paenibacillus endoradicis*	WP_258201581.1	UPI002159A85A	1082
98.	Bacteria	HYPO	GH13_48	*Clostridium thermarum*	WP_163195571.1	UPI0013D4B156	472
99.	Bacteria	HYPO	GH13_48	Flavobacteriales bacterium	RCL70465.1	A0A368DH14	446
100.	Bacteria	HYPO	GH13_48	Tenericutes bacterium ADurb.BinA124	OPZ32571.1	A0A1V5HM19	451
101.	Bacteria	HYPO	GH13_48	*Haloechinothrix* sp. LS1_15	WP_317493295.1	-	470
102.	Bacteria	HYPO	GH13_48	*Mucisphaera calidilacus*	QDU70590.1	A0A518BUD2	454
103.	Bacteria	HYPO	GH13_48	Bacteroidales bacterium 36-12	OJV31926.1	UPI00092C9222	451
104.	Bacteria	HYPO	GH13_48	*Empedobacter stercoris*	NOJ75523.1	UPI00149201F8	460
105.	Bacteria	HYPO	GH13_48	Verrucomicrobiota bacterium	RME68550.1	A0A3M1GIT3	461
106.	Bacteria	HYPO	GH13_48	*Pelagicoccus mobilis*	MBK1876700.1	A0A934RSC1	462
107.	Bacteria	HYPO	GH13_48	*Sediminibacterium* sp. TEGAF015	BDQ11811.1	UPI0021FA8CB5	422
108.	Bacteria	HYPO	GH13_48	Chitinophagaceae bacterium	RYY54105.1	A0A4Q5TU14	425
109.	Bacteria	HYPO	GH13_48	*Arachidicoccus ginsenosidivorans*	QEC72499.1	A0A5B8VMD8	452
110.	Bacteria	HYPO	GH13_48	Bacteroidota bacterium	TAF98278.1	-	422
111.	Bacteria	HYPO	GH13_48	Chitinophagaceae bacterium	PHX74369.1	A0A2G4GK25	443
112.	Bacteria	HYPO	GH13_48	Chitinophagaceae bacterium	RYY29886.1	A0A4Q5S6K0	427
113.	Bacteria	HYPO	GH13_48	*Flavihumibacter* sp. CACIAM 22H1	KYP14675.1	A0A162PPU5	431
114.	Bacteria	HYPO	GH13_48	*Niabella aurantiaca* DSM 17617	WP_018626150.1	UPI0003761C57	468
115.	Bacteria	HYPO	GH13_48	*Aridibaculum aurantiacum*	WP_207492959.1	UPI001A978631	429
116.	Bacteria	HYPO	GH13_48	Chitinophagaceae bacterium	RYY49171.1	A0A4Q5TGR9	436
117.	Bacteria	HYPO	GH13_48	*Ferruginibacter albus*	WP_224014770.1	UPI001CC3491E	431
118.	Bacteria	HYPO	GH13_48	*Sphingobium* sp. AP49	WHO38526.1	UPI00055A9BCD	469
119.	Bacteria	HYPO	GH13_48	*Litorimonas cladophorae*	GGX70803.1	A0A918KPA6	463
120.	Bacteria	HYPO	GH13_48	*Hirschia baltica* ATCC 49814	ACT60142.1	C6XNJ8	471
121.	Bacteria	HYPO	GH13_48	*Erythrobacter litoralis* HTCC 2594	ABC62721.1	Q2NC70	467
122.	Bacteria	HYPO	GH13_48	*Sphingomicrobium aestuariivivum*	WP_245112901.1	UPI001FD6E887	481
123.	Bacteria	HYPO	GH13_48	*Pseudoalteromonas* sp. HL-AS1	WMS92276.1	-	460
124.	Bacteria	HYPO	GH13_48	*Sphingomonas* sp. HMP9	BCA62694.1	A0A6J4BXU6	458
125.	Bacteria	HYPO	GH13_48	*Miniimonas arenae*	TNU76607.1	UPI000D52A23C	450
126.	Bacteria	HYPO	GH13_48	*Alteromonas macleodii* Black Sea 11	AFT78933.1	K0D290	434
127.	Bacteria	HYPO	GH13_48	*Thalassotalea eurytherma*	GLX81755.1	UPI00240B3E3B	470
128.	Bacteria	HYPO	GH13_48	*Thalassotalea mangrovi*	TKB44220.1	A0A4U1B3R9	442
129.	Bacteria	HYPO	GH13_48	*Chryseobacter iumgregarium* DSM 19109	WP_027386311.1	UPI00042130AC	441
130.	Bacteria	HYPO	GH13_48	Porphyromonadaceae bacterium	TSA33438.1	-	457
131.	Bacteria	HYPO	GH13_48	Bacteroidetes bacterium ADurb.Bin012	OQC56648.1	A0A1V6J256	447
132.	Bacteria	HYPO	GH13_48	Ignavibacteriales bacterium	QQS36543.1	UPI001AF8043D	557
133.	Bacteria	HYPO	GH13_48	Ignavibacteria bacterium	KAF0152141.1	A0A6A4UPQ1	572

134.	Bacteria	HYPO	GH13_48	Bacteroidota bacterium	QQS49654.1	UPI001AF5D40A	472
135.	Bacteria	HYPO	GH13_48	*Poriferisphaera corsica*	QDU34188.1	A0A517YVC6	480
136.	Bacteria	HYPO	GH13_48	*Spirochaeta africana* ATCC 700263	AFG37077.1	H9UHT4	478
137.	Bacteria	HYPO	GH13_48	Spirochaetes bacterium GWB1_27_13	OHD05762.1	A0A1G3KL75	465
138.	Bacteria	HYPO	GH13_48	bacterium	UCE04739.1	UPI001EA9C306	596
139.	Bacteria	HYPO	GH13_48	Bacteroidetes bacterium RBG_19FT_COMBO_42_10	OFY57429.1	A0A1F3MWF8	447
140.	Bacteria	HYPO	GH13_48	Bacteroidota bacterium	TNF42207.1	-	457
141.	Bacteria	HYPO	GH13_48	*Xylanibacter ruminicola* Ga6B6	WP_028906945.1	UPI0004914BB3	517
142.	Bacteria	HYPO	GH13_48	*Prevotella* sp. tf2-5	SFO44310.1	A0A1I5H8A1	461
143.	Bacteria	HYPO	GH13_48	Muribaculaceae bacterium	MDE5585204.1	UPI0023D5FC00	477
144.	Bacteria	HYPO	GH13_48	Paludibacteraceae bacterium	MCQ2351634.1	-	464
145.	Bacteria	HYPO	GH13_48	*Bacteroides xylanisolvens*	RHD66178.1	A0A414GBC1	454
146.	Bacteria	HYPO	GH13_48	Bacteroidales bacterium	MDR0619018.1	-	449
147.	Bacteria	HYPO	GH13_48	*Flavobacterium columnare*	PTD14489.1	A0A2T4HFA7	467
148.	Bacteria	HYPO	GH13_48	*Galbibacter mesophilus*	WP_202029432.1	UPI00191D084E	469
149.	Bacteria	HYPO	GH13_48	*Galbibacter mesophilus*	WP_202029447.1	UPI00191EBF4F	480
150.	Bacteria	HYPO	GH13_48	Lentisphaerota bacterium	RMD79510.1	A0A3M0Z5H8	499
151.	Bacteria	HYPO	GH13_48	Lentisphaerota bacterium	RMD82313.1	A0A3M0ZDV2	606
152.	Bacteria	HYPO	GH13_48	Abditibacteriota bacterium	BCM92162.1	UPI0020632D48	705
153.	Bacteria	HYPO	GH13_48	*Terrimonas ginsenosidimutans*	MCG2615063.1	UPI001EDBD405	452
154.	Bacteria	HYPO	GH13_48	*Psychrosphaera* sp. B3R10	WP_215963722.1	UPI001C0A57FD	473
155.	Bacteria	HYPO	GH13_48	*Hymenobacter swuensis* DY53	AHJ96057.1	W8EWB8	467
156.	Bacteria	HYPO	GH13_48	*Flavobacterium* sp. Root186	KRB54634.1	A0A0Q8N6W3	461
157.	Bacteria	HYPO	GH13_48	*Mucilaginibacter* sp. L294	WP_067058465.1	UPI00082E4143	453
158.	Bacteria	HYPO	GH13_48	*Marinigracilibium pacificum*	NMM49536.1	A0A848J147	455
159.	Archaea	HYPO	GH13_48	*Ferroplasma* sp.	WP_298409235.1	UPI00261A3EDE	440
160.	Bacteria	HYPO	GH13_48	*Pedobacter aquatilis*	WP_290243495.1	UPI0025B4FFD6	451
161.	Bacteria	HYPO	GH13_48	*Hymenobacter* sp. AT01-02	WP_052694997.1	UPI000697C007	464
162.	Bacteria	HYPO	GH13_48	*Flavobacterium* sp. CF108	SHH03180.1	A0A1M5PN57	1123
163.	Bacteria	HYPO	GH13_48	*Chryseobacterium ginsenosidimutans*	WP_259132588.1	UPI002169E3C0	741
164.	Bacteria	HYPO	GH13_48	*Dawidia soli*	WP_254089185.1	UPI0020B28B8E	871
165.	Bacteria	HYPO	GH13_48	*Fulvivirga ligni*	WP_233771144.1	UPI001F354002	937
166.	Bacteria	HYPO	GH13_48	*Flavobacterium* sp. AED	WP_082014116.1	UPI0009DEB5E1	687
167.	Bacteria	HYPO	GH13_48	*Hymenobacter* sp. PAMC 26628	AMJ67471.1	A0A126PH58	932
168.	Bacteria	HYPO	GH13_48	*Marinoscillum pacificum*	WP_258101097.1	UPI00215889A8	1145
169.	Bacteria	HYPO	GH13_48	*Reichenbachiella faecimaris*	SMD37960.1	A0A1W2GMR2	1339
170.	Bacteria	HYPO	GH13_48	*Mucilaginibacter gotjawali*	BAU52461.1	A0A110B104	461
171.	Bacteria	HYPO	GH13_48	*Bacteroides intestinalis*	RGV58439.1	A0A412YM05	447
172.	Bacteria	HYPO	GH13_48	*Lascolabacillus massiliensis*	WP_053826811.1	UPI0006B38A97	451
173.	Bacteria	HYPO	GH13_48	Uncultured *Phocaeicola*sp.	WP_294586801.1	-	445
174.	Bacteria	HYPO	GH13_48	*Bacteroides uniformis*3978 T3 ii	KDS55067.1	A0A078S3D5	419
175.	Bacteria	HYPO	GH13_48	Muribaculaceae bacterium	GFI38810.1	A0A7J0AP65	447
176.	Bacteria	HYPO	GH13_48	*Psychrosphaera* sp. I2R16	WP_215963728.1	UPI001C07F396	921
177.	Bacteria	HYPO	GH13_48	*Bythopirellula goksoeyrii*	QEG34451.1	A0A5B9QJW6	517
178.	Bacteria	HYPO	GH13_48	Phycisphaeraceae bacterium	USO00269.1	UPI0022080067	704
179.	Bacteria	HYPO	GH13_48	Sphingobacteriales bacterium BACL12 MAG-120802-bin5	KRP08913.1	A0A0R2VID0	438

180.	Bacteria	HYPO	GH13_48	*Flavobacterium* sp. UBA6135	WP_291115335.1	-	442
181.	Bacteria	HYPO	GH13_48	*Flaviaesturariibacter aridisoli*	TCZ69633.1	A0A4R4DZE9	455
182.	Bacteria	HYPO	GH13_48	*Polaribacter atrinae*	OAD45612.1	A0A176TCL6	456
183.	Bacteria	HYPO	GH13_48	bacterium	TNE71538.1	-	457
184.	Bacteria	HYPO	GH13_48	*Croceimicrobium hydrocarbonivorans*	QNR24262.1	A0A7H0VEW4	462
185.	Bacteria	HYPO	GH13_48	*Carboxylicivirga* sp. A043	WP_262325912.1	UPI0021CB7CA0	457
186.	Bacteria	HYPO	GH13_48	*Parachryseolinea silvisoli*	WP_267292534.1	UPI002265E23A	455
187.	Bacteria	HYPO	GH13_48	Ignavibacteriae bacterium HGW-Ignavibacteriae-2	PKL87522.1	A0A2N1VSV7	462
188.	Bacteria	HYPO	GH13_48	*Microbacter margulisiae*	MBB3187405.1	A0A7W5DQU4	450
189.	Bacteria	MGA	GH13_48	*Uncultured bacterium	QYD13596.1	-	477
190.	Bacteria	HYPO	GH13_48	*Bacteroides stercorirosoris*	WP_259321779.1	UPI00216B09EA	455
191.	Bacteria	HYPO	GH13_48	*Segatella buccae*	EFU30965.1	E6K618	450
192.	Bacteria	HYPO	GH13_48	*Reichenbachiella faecimaris*	WP_084374187.1	UPI000A042008	454
193.	Bacteria	HYPO	GH13_48	*Marinoscillum pacificum*	WP_258101092.1	UPI00215818FE	449
194.	Bacteria	HYPO	GH13_48	*Fulvivirga ligni*	WP_233771145.1	UPI001F1C11D5	453
195.	Bacteria	HYPO	GH13_48	*Marinilabilia* sp.	WP_291857032.1	-	462
196.	Bacteria	HYPO	GH13_48	*Plebeiobacter iumsediminum*	WP_301189372.1	UPI00263AC6E5	470
197.	Bacteria	HYPO	GH13_48	Bacteroidales bacterium	PCH71087.1	A0A2A4NGC4	488
198.	Bacteria	HYPO	GH13_48	*Indibacter alkaliphilus* LW1	WP_160169343.1	UPI0013640F1D	464
199.	Bacteria	HYPO	GH13_48	*Algoriphagus* sp. NG3	WPR76056.1	-	462
200.	Bacteria	HYPO	GH13_48	*Hymenobacter* sp. BT770	WP_262907729.1	UPI0021D44583	435
201.	Bacteria	HYPO	GH13_48	*Flexibacter flexilis* DSM 6793	SFC52663.1	A0A1I1K3S7	459
202.	Bacteria	HYPO	GH13_48	Gemmatimonadota bacterium	PYO13003.1	A0A2V7EL81	458
203.	Bacteria	HYPO	GH13_48	bacterium	RYG26828.1	A0A4Q3UK90	448
204.	Bacteria	HYPO	GH13_48	Elusimicrobia bacterium CG08_land_8_20_14_0_20_44_26	PIU18304.1	A0A2M6Y062	471
205.	Bacteria	HYPO	GH13_48	*Salinispira pacifica*	AHC16151.1	V5WM22	475
206.	Bacteria	HYPO	GH13_48	Bacteroidota bacterium	GDX48839.1	A0A4P5U7M4	415
207.	Bacteria	HYPO	GH13_48	Candidatus *Thermochlorobacter aerophilum*	RFM23311.1	A0A395LXW5	470
208.	Bacteria	HYPO	GH13_48	Planctomycetaceae bacterium	CAG0933593.1	A0A916BUJ2	609
209.	Bacteria	HYPO	GH13_48	Bacteroidota bacterium	KAB2921026.1	A0A7J5F0F1	506
210.	Bacteria	HYPO	GH13_48	Ignavibacteria bacterium GWC2_56_12	OGU46553.1	UPI0008BC994B	459
211.	Bacteria	HYPO	GH13_48	Phycisphaerales bacterium	UCF34196.1	UPI001EA98AA6	583
212.	Bacteria	HYPO	GH13_48	Calditrichota bacterium	KAA3610944.1	A0A6H9JZ97	589
213.	Bacteria	HYPO	GH13_48	Verrucomicrobiota bacterium	PWU13395.1	A0A2V2S098	471
214.	Bacteria	HYPO	GH13_48	*Nevskia soli*	WP_180538988.1	UPI0015D82BBC	417
215.	Bacteria	HYPO	GH13_48	*Edaphobacter aggregans* DSM 19364	WP_051978849.1	UPI000691AADF	500
216.	Bacteria	HYPO	GH13_48	*Capsulimonas corticalis*	WP_119321018.1	UPI000E656AE2	457
217.	Bacteria	HYPO	GH13_48	*Capsulimonas corticalis*	BDI33655.1	A0A402D0B3	484
218.	Eucarya	HYPO	GH13_48	*Letharia columbiana*	KAF6232848.1	A0A8H6FQI5	614
219.	Bacteria	HYPO	GH13_48	*Granulicella tundricola ATCC BAA-1859*	ADW71132.1	E8X723	489
220.	Bacteria	HYPO	GH13_48	*Deinococcus misasensis* DSM 22328	WP_051963117.1	UPI0006897FEA	431
221.	Bacteria	HYPO	GH13_48	Bryobacterales bacterium F-183	BDC50083.1	-	398
222.	Bacteria	HYPO	GH13_48	Candidatus Marinimicrobia bacterium CG08	PIS27281.1	A0A2H0XR00	609
223.	Bacteria	HYPO	GH13_48	candidate division KSB1 bacterium 4484_188	OPX33725.1	A0A1V4RRH5	395
224.	Bacteria	HYPO	GH13_48	*Caldithrix abyssi* DSM 13497	APF16921.1	H1XTZ1	464
225.	Bacteria	HYPO	GH13_48	Calditrichota bacterium	RMF58283.1	A0A3M1NU83	467

226.	Bacteria	HYPO	GH13_48	Gemmatimonadota bacterium	UCE20243.1	UPI001EAC6FE6	469
227.	Bacteria	HYPO	GH13_48	*Heliomicrobium undosum*	MZP29388.1	A0A845L6L4	523
228.	Bacteria	HYPO	GH13_48	Spirochaetes bacterium GWF1_51_8	OHD53505.1	A0A1G3PGI9	535
229.	Bacteria	HYPO	GH13_48	*Caldithrix abyssi* DSM 13497	APF16786.1	H1XUP9	441
230.	Bacteria	HYPO	GH13_48	Calditrichota bacterium	NOG46902.1	A0A849MAX3	454
231.	Bacteria	HYPO	GH13_48	*Lactobacillus delbrueckii* DSM 26046	APG72266.1	UPI00032F5892	429
232.	Bacteria	HYPO	GH13_48	*Porcincola intestinalis*	MSS14465.1	A0A6L5X4C2	460
233.	Bacteria	HYPO	GH13_48	*Lactobacillus* sp. CBA3605	AVK61635.1	A0A2R3JPU4	426
234.	Bacteria	HYPO	GH13_48	Uncultured *Ruminococcus sp.*	WP_316608594.1	-	460
235.	Bacteria	HYPO	GH13_48	Spirochaetia bacterium	GHV84474.1	UPI00221D3270	454
236.	Bacteria	HYPO	GH13_48	*Kineothrix alysoides*	TCL61159.1	A0A4R1R6R4	431
237.	Bacteria	HYPO	GH13_48	*Alkaliphilus transvaalensis* ATCC 700919	WP_026477530.1	UPI000479CE79	431
238.	Bacteria	HYPO	GH13_48	Gottschalkiaceae bacterium SANA	BES65512.1	-	429
239.	Bacteria	HYPO	GH13_48	*Globicatella sanguinis*	PKZ43968.1	A0A2I1PH64	424
240.	Bacteria	HYPO	GH13_48	*Enterococcus* sp. 3H8_DIV0648	OTO19165.1	A0A242D9R4	425
241.	Bacteria	HYPO	GH13_48	*Clostridium thermarum*	WP_163194646.1	UPI0013D465BD	429
242.	Bacteria	HYPO	GH13_48	*Dolosicoccus paucivorans*	PMC58021.1	A0A2N6SLS0	432
243.	Bacteria	HYPO	GH13_48	*Facklamia miroungae*	SDG31851.1	A0A1G7T9F5	431
244.	Archaea	HYPO	GH13_48	*Thermococcus* sp. 2319x1	WP_175058779.1	UPI0015835AED	414
245.	Archaea	HYPO	GH13_48	*Palaeococcus ferrophilus* DSM 13482	WP_048148786.1	UPI00064F8900	411
246.	Bacteria	HYPO	GH13_48	Firmicutes bacterium CAG:129	CCZ46699.1	R5S796	428
247.	Bacteria	HYPO	GH13_48	*Companilactobacillus bobalius*	OVE98933.1	A0A202FEQ7	430
248.	Bacteria	HYPO	GH13_48	*Desemzia* sp. C1	WP_230523982.1	UPI001E51B6DD	457
249.	Bacteria	HYPO	GH13_48	Uncultured *Acetatifactor* sp.	WP_300788324.1	UPI002639E2EF	443
250.	Bacteria	HYPO	GH13_48	*Salana multivorans*	ROR96918.1	A0A3N2DB81	439
251.	Bacteria	HYPO	GH13_48	*Paracholeplasma vituli*	WP_262096796.1	UPI0021C9EBB6	432
252.	Archaea	HYPO	GH13_48	*Methanobacterium* sp. YSL	MBW4258460.1	-	432
253.	Bacteria	HYPO	GH13_48	*Hujiaoplasma nucleasis*	QLY40718.1	A0A7L6N645	427
254.	Bacteria	HYPO	GH13_48	Tenericutes bacterium GWF2_57_13	OHE40447.1	A0A1G3WJX7	429
255.	Bacteria	HYPO	GH13_48	*Acholeplasma equifetale* ATCC 29724	WP_026399499.1	UPI00047A6BA7	425
256.	Bacteria	HYPO	GH13_48	*Turicibacter* sp. TC023	BEH89920.1	-	436
257.	Bacteria	HYPO	GH13_48	*Treponema* sp. GWC1_61_84	OHE60741.1	A0A1G3YP68	429
258.	Bacteria	HYPO	GH13_48	Uncultured Spirochaetota bacterium	VBB38884.1	A0A652ZSX9	450
259.	Bacteria	HYPO	GH13_48	Spirochaetes bacterium RIFOXYC1_FULL_54_7	OHD80806.1	A0A1G3RPE9	431
260.	Bacteria	HYPO	GH13_48	Tenericutes bacterium ADurb.Bin239	OQA78072.1	A0A1V5UGN4	427
261.	Bacteria	HYPO	GH13_48	Firmicutes bacterium HGW-Firmicutes-10	PKM90342.1	A0A2N2E6S9	430
262.	Eucarya	HYPO	GH13_48	*Pelomyxa schiedti*	KAH3742584.1	-	379
263.	Eucarya	HYPO	GH13_48	*Pelomyxa schiedti*	KAH3743471.1	-	542
264.	Bacteria	HYPO	GH13_48	*Faecalibaculum rodentium*	AMK53440.1	A0A140DS13	468
265.	Eucarya	HYPO	GH13_48	*Rhodosorus marinus*	KAJ8907911.1	UPI0024976C6B	453
266.	Bacteria	HYPO	GH13_48	*Leifsonia poae*	WP_223695379.1	UPI001CC02A5E	449
267.	Bacteria	HYPO	GH13_48	Firmicutes bacterium ADurb.Bin153	OQB49003.1	A0A1V6A978	434
268.	Bacteria	HYPO	GH13_48	Deltaproteobacterium ML8_F1	OPL08713.1	A0A1V4M218	419
269.	Bacteria	HYPO	GH13_48	*Spirochaeta cellobiosiphila* DSM 17781	WP_053228273.1	UPI00041BF7F2	422
270.	Eucarya	HYPO	GH13_48	*Rotaria* sp. Silwood1	CAF4711217.1	A0A821J158	1048
271.	Eucarya	HYPO	GH13_48	*Adineta steineri*	CAF1134171.1	A0A816B4B9	468

272.	Eucarya	HYPO	GH13_48	*Didymodactylos carnosus*	CAF1102446.1	A0A814P7F0	474
273.	Bacteria	HYPO	GH13_48	Chloroflexi bacterium RBG_19FT_COMBO_47_9	OGO61108.1	A0A1F8SML8	446
274.	Bacteria	HYPO	GH13_48	*Streptococcus suis*	MDG4518168.1	UPI001C986287	437
275.	Bacteria	HYPO	GH13_48	*Paenibacillus montanisoli*	RAP77929.1	A0A328U5U3	423
276.	Bacteria	HYPO	GH13_48	*Olavius algarvensis*	VDB00751.1	UPI000F0FF0DC	473
277.	Bacteria	HYPO	GH13_48	*Petrotoga mobilis* DSM 10674	ABX32406.1	A9BIB5	426
278.	Bacteria	HYPO	GH13_48	*Galactobacillus timonensis*	WP_276826105.1	UPI000EEC0286	422
279.	Bacteria	HYPO	GH13_48	*Stecheria intestinalis*	WP_277085470.1	UPI0023F08600	422
280.	Bacteria	HYPO	GH13_48	*Holdemania massiliensis* AP2	WP_020225392.1	UPI0002E132A5	435
281.	Bacteria	HYPO	GH13_48	*Erysipelothrix urinaevulpis*	WP_159519015.1	UPI0013576815	428
282.	Bacteria	HYPO	GH13_48	*Aerococcus christensenii*	KXB34120.1	A0A133XT65	434
283.	Bacteria	HYPO	GH13_48	*Clostridium* sp. CAG:568	CDA37557.1	UPI00033C5448	436
284.	Bacteria	HYPO	GH13_48	*Treponema rectale*	QOS39137.1	A0A7M1XIH0	425
285.	Bacteria	HYPO	GH13_48	Erysipelotrichales bacterium	TFG82036.1	A0A524K766	436
286.	Bacteria	HYPO	GH13_48	*Anaeroplasma bactoclasticum*	RIA78466.1	A0A397S112	430
287.	Bacteria	HYPO	GH13_48	Firmicutes bacterium CAG:345	CDD23883.1	R6XJV2	433
288.	Bacteria	MGA	GH13_48	**Thermotoga neapolitana*	ACF75909.1	B5ARZ9	422
289.	Bacteria	MGA	GH13_48	**Thermotoga maritima* ATCC 43589	AAD36717.1	Q9X1Y3	422
290.	Bacteria	MGA	GH13_48	**Lactiplantibacillus plantarum* WCFS1	ADN97370.1	F9USZ1	440
291.	Bacteria	HYPO	GH13_48	*Mesoaciditoga lauensis* DSM 25116	WP_051962564.1	UPI000691295A	420
292.	Bacteria	HYPO	GH13_48	Thermotogales bacterium 46_20	KUK93804.1	A0A117MAS0	427
293.	Bacteria	HYPO	GH13_48	bacterium 3DAC	UZN23494.1	-	419
294.	Bacteria	HYPO	GH13_48	Victivallales bacterium CCUG 44730	AVM44639.1	A0A2S0KUH4	443
295.	Bacteria	HYPO	GH13_48	*Coraliomargarita* sp. CAG:312	CDE85188.1	R7LB60	468
296.	Bacteria	HYPO	GH13_48	Opitutaceae bacterium TAV1	EIQ00463.1	I6AZM1	454
297.	Bacteria	HYPO	GH13_48	Lentisphaerae bacterium RIFOXYB12_FULL_65_16	OGV75364.1	A0A1G1BY54	428
298.	Bacteria	HYPO	GH13_48	Bacteroidetes bacterium GWF2_42_66	OFY42124.1	A0A1F3LNF5	463
299.	Bacteria	HYPO	GH13_48	*Oligosphaera ethanolica*	MDQ0291296.1	UPI0027835D74	466
300.	Bacteria	HYPO	GH13_48	Verrucomicrobia bacterium ADurb.Bin070	OQC30811.1	A0A1V6GY83	663
301.	Bacteria	HYPO	GH13_48	Verrucomicrobiota bacterium	PWL66745.1	A0A2V2BWL4	495
302.	Bacteria	HYPO	GH13_48	Verrucomicrobia bacterium GWF2_62_7	OHE78978.1	A0A1G3ZQ46	472
303.	Bacteria	HYPO	GH13_48	Opitutaceae bacterium TAV1	EIQ01573.1	I6B2T1	453
304.	Bacteria	HYPO	GH13_48	Acidobacteriota bacterium	PYV47674.1	A0A2V9N241	509
305.	Bacteria	HYPO	GH13_38	*Flavobacterium johnsoniae*	ABQ05620.1	A5FGP4	610
306.	Bacteria	AGLU	GH13_38	*Saccharophagus degradans*	ABD79820.1	Q21NA9	624
307.	Bacteria	AGLU	GH13_38	*Bacteroides cellulosilyticus*	ALJ62728.1	A0A0P0GJC2	565
308.	Archaea	HYPO	GH13_49	*Halapricum salinum*	WP_049993719.1	A0A4D6H8B6	1792
309.	Archaea	HYPO	GH13_49	*Halapricum salinum*	QCC51754.1	A0A4D6HCF1	1585
310.	Archaea	HYPO	GH13_49	*Halapricum* sp. CBA1109	MUV89328.1	A0A6A9SVZ3	1657
311.	Archaea	HYPO	GH13_49	*Halalkalirubrum salinum*	WP_138006871.1	UPI0010FB5E24	1323
312.	Archaea	HYPO	GH13_49	*Halapricum* sp. CBA1109	MUV89327.1	A0A6A9SYW8	1599
313.	Archaea	HYPO	GH13_49	*Halapricum desulfuricans*	QSG09447.1	A0A897N9R8	745
314.	Archaea	HYPO	GH13_49	*Natronoarchaeum rubrum*	WP_256393162.1	UPI0021123815	1316
315.	Archaea	HYPO	GH13_49	*Natranaeroarchaeum sulfidigenes*	QSG01523.1	A0A897MR85	1002
316.	Archaea	HYPO	GH13_49	*Natronococcus* sp. A-GB1	MDG5757732.1	UPI00241D3612	1347
317.	Archaea	HYPO	GH13_49	*Natranaeroarchaeum sulfidigenes*	QSG01730.1	A0A897MHW9	1400

318.	Archaea	HYPO	GH13_49	*Natronosalvus amylolyticus*	WP_254808009.1	-	996
319.	Archaea	HYPO	GH13_49	*Halobellus litoreus*	WP_256307440.1	UPI00210ED890	841
320.	Archaea	HYPO	GH13_49	*Natranaeroarchaeum sulfidigenes*	QSG04317.1	A0A897MV03	1346
321.	Archaea	HYPO	GH13_49	*Halapricum salinum*	QCC50410.1	A0A4D6H9E6	1618
322.	Archaea	HYPO	GH13_49	*Halomicroarcula marina*	WP_254279402.1	UPI0020B736EA	1210
323.	Archaea	HYPO	GH13_49	*Natrialba chahannaoensis* JCM 10990	ELY98223.1	M0ALG5	764
324.	Archaea	HYPO	GH13_49	*Natronoarchaeum rubrum*	WP_256392141.1	-	723
325.	Archaea	HYPO	GH13_49	*Natronoarchaeum philippinense*	SNZ15029.1	A0A285NZZ7	698
326.	Archaea	HYPO	GH13_49	*Halopiger aswanensis*	RKD95873.1	A0A419WK94	958
327.	Archaea	HYPO	GH13_49	*Natronoarchaeum rubrum*	WP_256390983.1	-	890
328.	Archaea	AAMY	GH13_49	**Haloferax alexandrinus*	QIB80089.1	A0A6C0V1X1	668
329.	Archaea	HYPO	GH13_49	*Haloferax mucosum*ATCC BAA-1512	ELZ98706.1	M0IPF3	683
330.	Archaea	HYPO	GH13_49	*Halorubrum tebenquichense*DSM 14210	ELZ35579.1	M0DN69	701
331.	Archaea	HYPO	GH13_49	*Haloarchaeobius amylolyticus*	WP_267643473.1	UPI002270A13E	673
332.	Archaea	HYPO	GH13_49	*Halococcoides cellulosivorans*	AWB27109.1	A0A2R4X015	643
333.	Archaea	HYPO	GH13_49	Halobacteriales archaeon QS_6_71_20	PSQ08025.1	A0A2R6IV27	687
334.	Archaea	AAMY	GH13_49	**Haloarcula japonica* ATCC 49778	BAM75337.1	L8B068	663
335.	Archaea	HYPO	GH13_49	*Halapricum desulfuricans*	QSG15657.1	A0A897NS85	634
336.	Archaea	HYPO	GH13_49	*Natronosalvus amylolyticus*	WP_254808560.1	UPI0020C97C11	631
337.	Archaea	HYPO	GH13_49	uncultured archaeon A07HN63	ESS08848.1	V4Y2C1	776
338.	Archaea	HYPO	GH13_49	*Salinirubrum litoreum*	WP_227229223.1	UPI001D097583	781
339.	Archaea	HYPO	GH13_49	*Halobaculum saliterrae*	MXR40679.1	A0A6B0T2C0	792
340.	Archaea	HYPO	GH13_49	*Halorubrum terrestre* JCM 10247	ELZ34269.1	M0DJB8	783
341.	Archaea	HYPO	GH13_49	*Natronosalvus vescus*	WP_252700766.1	UPI00208FFCA3	721
342.	Archaea	HYPO	GH13_49	*Natronoarchaeum philippinense*	SNZ12752.1	A0A285NTC8	685
343.	Archaea	HYPO	GH13_49	*Haloprofundus marisrubri*	KTG11576.1	A0A0W1RDT8	700
344.	Archaea	HYPO	GH13_49	*Halomicroarcula marina*	WP_254272790.1	UPI0020B7BADD	1108
345.	Archaea	HYPO	GH13_49	*Halapricum salinum*	QCC50471.1	A0A4D6HAB4	716
346.	Bacteria	HYPO	GH13_49	bacterium BMS3Abin05	GBD94596.1	A0A2H6EYQ1	945
347.	Bacteria	HYPO	GH13_49	candidate division KSB1 bacterium 4572_119	OQX94954.1	A0A1W9SDQ2	929
348.	Bacteria	HYPO	GH13_49	*Caldithrix abyssi*DSM 13497	APF20939.1	H1XVA1	874
349.	Bacteria	HYPO	GH13	bacterium BMS3Bbin03	GBE26813.1	UPI000CC329BE	1396
350.	Bacteria	HYPO	GH13	Gemmatimonadota bacterium	UCE20242.1	UPI001EA9C2E7	1284
351.	Bacteria	GPMT	GH13_3	*Mycobacterium tuberculosis*	CCP44085.1	P9WQ17	701
352.	Bacteria	GPMT	GH13_3	*Mycolicibacterium thermoresistibile*	EHI11807.1	G7CL00	696
353.	Bacteria	GPMT	GH13_3	*Streptomyces coelicolor*	CAB72419.1	Q9L1K2	675
354.	Eucarya	GDE	GH13_25	*Homo sapiens*	AAB41040.1	P35573	1532
355.	Eucarya	GDE	GH13_25	*Oryctolagus cuniculus*	AAA16364.1	P35574	1555
356.	Eucarya	GDE	GH13_25	*Candida glabrata*	CAG59721.1	Q6FSK0	1528
357.	Bacteria	GDGE	GH13_12	*Streptococcus agalactiae*	AAN00098.1	Q8DZ94	1252
358.	Bacteria	PUL	GH13_12	*Streptococcus pyogenes*	CAD32942.1	Q8KLP1	1165
359.	Bacteria	PUL	GH13_12	*Streptococcus pneumoniae*	AAK74446.1	A0A0H2UNG0	1280
360.	Eucarya	LDE	GH13_13	*Hordeum vulgare*	AAD04189.1	O48541	904
361.	Eucarya	PUL	GH13_13	*Zea mays*	AAD11599.1	O81638	962
362.	Bacteria	PUL	GH13_13	*Klebsiella pneumoniae*	CAA36431.1	P07206	1090
363.	Bacteria	PUL	GH13_14	*Anoxybacillus* sp. LM18-11	AEW23439.1	K9L0H1	707

364.	Bacteria	PUL	GH13_14	*Paenibacillus barengoltzii*	AJP16551.1	A0A0C5GWS2	675
365.	Bacteria	PUL	GH13_14	*Thermotoga maritima*	CAA04522.1	O33840	843
366.	Eucarya	ISA	GH13_11	*Chlamydomonas reinhardtii*	AAP85534.1	Q7X8Q2	875
367.	Archaea	ISA	GH13_11	*Sulfolobus acidocaldarius*	ALU30386.1	O05152	713
368.	Bacteria	ISA	GH13_11	*Pseudomonas amyloderamosa*	CAA31754.1	P10342	776
369.	Bacteria	MOTH	GH13_10	*Kocuria rosea*	AAY83363.1	Q4JQI8	624
370.	Archaea	MOTH	GH13_10	*Saccharolobus solfataricus*	BAA11010.1	Q55088	559
371.	Bacteria	MOTH	GH13_10	*Deinococcus radiodurans*	AAF10042.1	Q9RX51	600
372.	Bacteria	HYPO	GH13_47	*Flavobacterium indicum* DSM 17447	CCG52206.1	H8XNT9	947
373.	Bacteria	HYPO	GH13_47	*Phocaeicola vulgatus*274-1D4	WP_100263035.1	UPI000C21E611	859
374.	Bacteria	AAMY	GH13_47	*Bacteroides ovatus*ATCC 8483	ALJ48408.1	A7M087	758
375.	Eucarya	GBE	GH13_8	*Homo sapiens*	AAA58642.1	Q04446	702
376.	Eucarya	GBE	GH13_8	*Oryza sativa*	BAA01584.1	Q01401	820
377.	Eucarya	GBE	GH13_8	*Aspergillus oryzae*	BAB69770.1	Q96VA4	689
378.	Bacteria	GBE	GH13_9	*Crocosphaera subtropica*	ACB51598.1	B1WPM8	773
379.	Bacteria	GBE	GH13_9	*Escherichia coli*	AAA23872.1	P07762	728
380.	Bacteria	GBE	GH13_9	*Rhodothermus marinus*	BAB69858.1	Q93HU3	621
381.	Eucarya	AAMY	GH13_24	*Gallus gallus*	AAC60246.1	Q98942	512
382.	Eucarya	AAMY	GH13_24	*Homo sapiens* (saliva)	AAH63129.1	P04745	511
383.	Eucarya	AAMY	GH13_24	*Sus scrofa* (pancreas)	AAF02828.1	P00690	511
384.	Eucarya	AAMY	GH13_15	*Tenebrio molitor*	-	P56634	471
385.	Eucarya	AAMY	GH13_15	*Tribolium castaneum*	AAA03708.1	Q26854	490
386.	Eucarya	AAMY	GH13_15	*Drosophila melanogaster*	AAA92226.1	P08144	494
387.	Bacteria	AAMY	GH13_32	*Microbacterium aurum* (AmyA)	AKG25402.1	A0A0G2T4B5	1417
388.	Bacteria	AAMY	GH13_32	*Streptomyces limosus*	AAA88554.1	P09794	566
389.	Bacteria	AAMY	GH13_32	*Pseudoalteromonas haloplanktis*	CAA41481.1	P29957	668
390.	Bacteria	AAMY	GH13_28	*Bacillus subtilis*	CAA30643.1	Q45520	477
391.	Bacteria	AAMY	GH13_28	*Lactobacillus amylovorus*	ABO77965.1	A4ULJ3	478
392.	Bacteria	AAMY	GH13_28	*Clostridium acetobutylicum*	AAA63759.2	P23671	760
393.	Bacteria	AAMY	GH13_27	*Pseudomonas* sp. KFCC10818	AAA86836.1	Q52414	466
394.	Bacteria	AAMY	GH13_27	*Xanthomonas campestris*	AAA27591.1	Q56791	475
395.	Bacteria	AAMY	GH13_27	*Aeromonas hydrophila*	AAA21936.1	P22630	464
396.	Archaea	HYPO	GH13_43	*Haladaptatus paucihalophilus*	EFW93629.1	E7QNX6	404
397.	Archaea	M3H	GH13_43	*Natronococcus sp. Ah-36*	BAA05516.1	Q60224	504
398.	Archaea	AAMY	GH13_43	*Haloarcula hispanica*	CAI64586.1	Q4A3E0	433
399.	Eucarya	AAMY	GH13_6	*Hordeum vulgare*	AAA32929.1	P00693	438
400.	Eucarya	AAMY	GH13_6	*Oryza sativa*	AAA33885.1	P17654	434
401.	Bacteria	M6H	GH13_6	*Corallococcus sp. EGB*	AII00648.1	A0A076EBZ6	522
402.	Archaea	AAMY	GH13_7	*Pyrococcus woesei*	AAD54338.1	Q7LYT7	460
403.	Bacteria	AAMY	GH13_7	*Sinomicrobium* sp. 5DNS001	AGD88873.1	L7Y1I6	478
404.	Archaea	AAMY	GH13_7	*Thermococcus hydrothermalis*	AAC97877.1	O93647	457
405.	Bacteria	AAMY	GH13_5	*Alicyclobacillus* sp. 18711	AWX66236.1	A0A3P8MUS3	514
406.	Bacteria	AAMY	GH13_5	*Bacillus amyloliquefaciens*	AAA22191.1	P00692	514
407.	Bacteria	AAMY	GH13_5	*Halothermothrix orenii* (AmyB)	ACL70573.1	B8CZ54	623
408.	Eucarya	AAMY	GH13_1	*Aspergillus oryzae*	CAA31218.1	P0C1B3	499
409.	Eucarya	AAMY	GH13_1	*Saccharomycopsis fibuligera*	ADD80242.1	D4P4Y7	494

410.	Eucarya	AAMY	GH13_1	*Lipomyces kononenkoae*	AAO12212.1	Q8J1E4	499
411.	Bacteria	M6H	GH13_42	*Microbacterium aurum* (AmyB)	AOF40721.1	A0A1B3IKE0	1278
412.	Bacteria	AAMY	GH13_42	*Streptomyces lividans*	CAB06816.1	P96992	993
413.	Bacteria	HYPO	GH13_42	*Cystobacter fuscus*	AAW03335.1	Q5MD26	978
414.	Bacteria	AAMY	GH13_19	*Escherichia coli*	CAA41740.1	P25718	676
415.	Bacteria	M6H	GH13_19	*Klebsiella pneumoniae*	BAA88434.1	Q9RHR1	677
416.	Bacteria	M6H	GH13_19	*Bacillus halodurans*	BAB04132.1	Q9KFR4	958
417.	Bacteria	AAMY	GH13_41	*Eubacterium rectale*	CBK91127.1	D6DYI9	1364
418.	Bacteria	AAMY	GH13_41	*Roseburia* sp. A2-194	CAJ20070.1	Q3LB10	1674
419.	Bacteria	AAMY	GH13_41	*Micrococcus* sp. 207	CAA39321.1	Q06812	1104
420.	Bacteria	CGT	GH13_2	*Anaerobranca gottschalkii*	CAH61550.1	Q5ZEQ7	721
421.	Bacteria	CGT	GH13_2	*Bacillus circulans*	CAA55023.1	P43379	713
422.	Bacteria	MGAA	GH13_2	*Geobacillus stearothermophilus*	AAA22233.1	P19531	719
423.	Bacteria	AAMY	GH13_45	*Anoxybacillus* sp. SK3_4	JF932307.1	I1VWH9	505
424.	Bacteria	AAMY	GH13_45	*Priestia megaterium* (BmaN1)	AGT45938.1	T1SIF2	504
425.	Bacteria	AAMY	GH13_45	*Bacillus aquimaris* (BaqA)	AER68125.1	G8IJA7	512
426.	Bacteria	NPUL	GH13_46	*Bacteroides thetaiotaomicron*(SusA)	CAD32957.1	Q8A1G0	617
427.	Bacteria	AAMY	GH13_46	*Zunongwangia profunda*	ADF53136.1	D5BG23	615
428.	Bacteria	CMD	GH13_46	*Flavobacterium* sp. No. 92	AAO78809.1	Q8KKG0	619
429.	Eucarya	AGS	GH13_22	*Cryptococcus neoformans*	AAW44814.1	Q5KDD1	2430
430.	Eucarya	AGS	GH13_22	*Schizosaccharomyces pombe*	BAA76558.1	Q9UUL4	2352
431.	Eucarya	AGS	GH13_22	*Neosartorya fumigata*	AAL28129.1	Q96UQ6	2420
432.	Bacteria	CMD	GH13_20	*Bacillus* sp. I-5	AAA92925.1	Q59226	558
433.	Bacteria	MGA	GH13_20	*Thermus sp. IM6501*	AAC15072.1	O69007	588
434.	Bacteria	NPUL	GH13_20	*Thermoactinomyces vulgaris* (TVAII)	BAA02473.1	Q08751	585
435.	Bacteria	APUL	GH13_39	*Alicyclobacillus acidocaldarius*	ACV59878.1	C8WUR2	1299
436.	Bacteria	APUL	GH13_39	*Geobacillus thermoleovorans*	AFI70750.1	I1WWV6	1655
437.	Bacteria	APUL	GH13_39	*Thermoanaerobacter thermohydrosulfuricus*	AAA23205.1	P16950	1475
438.	Bacteria	HYPO	GH13_21	*Edwardsiella tarda*	ACY83800.1	D0ZE69	596
439.	Bacteria	AGLU	GH13_21	*Escherichia coli*	CAA42498.1	P21517	604
440.	Bacteria	AAMY	GH13_21	*Thermoactinomyces vulgaris* (TVAI)	BAA02471.1	Q60053	666
441.	Bacteria	HYPO	GH13_37	*Aliivibrio fischeri*	AAW86764.1	Q5E2I2	634
442.	Bacteria	AAMY	GH13_37	Uncultured bacterium	ADK21254.1	D9MZ14	638
443.	Bacteria	HYPO	GH13_37	*Hahella chejuensis*	ABC33052.1	Q2S8H2	556
444.	Bacteria	SPH	GH13_18	*Bifidobacterium adolescentis*	AAO33821.1	A0ZZH6	504
445.	Bacteria	SPH	GH13_18	*Streptococcus mutans*	CAA30846.1	P10249	481
446.	Bacteria	SPH	GH13_18	*Thermoanaerobacterium thermosaccharolyticum*	ADL69407.1	D9TT09	488
447.	Archaea	MOTS	GH13_26	*Saccharolobus shibatae*	AAF17554.1	Q9UWN8	728
448.	Archaea	MOTS	GH13_26	*Sulfolobus acidocaldarius*	ALU30387.1	Q53688	720
449.	Bacteria	MOTS	GH13_26	*Rhizobium* sp. M-11	BAA11186.1	Q53237	772
450.	Bacteria	HYPO	GH13_33	*Kineococcus radiotolerans*	ABS05210.1	A6WEH1	774
451.	Bacteria	HYPO	GH13_33	*Rhodococcus jostii*	ABG96354.1	Q0S7Y2	814
452.	Bacteria	TSY	GH13_33	*Pseudomonas stutzeri*	AAF26837.1	Q9LAS5	689
453.	Eucarya	4F2hc	GH13_34	*Homo sapiens* (isoform 1)	AAA35489.1	P08195	630
454.	Eucarya	4F2hc	GH13_34	*Mus musculus*	AAA35489.1	P10852	526
455.	Eucarya	4F2hc	GH13_34	*Salmo salar*	ACI33885.1	B5X3K4	510

456.	Bacteria	OGLU	GH13_31	*Bacillus cereus*	CAA37583.1	P21332	558
457.	Bacteria	SIM	GH13_31	*Pseudomonas mesoacidophila*	ABC33903.1	Q2PS28	584
458.	Bacteria	DGLU	GH13_31	*Streptococcus mutans*	BAE79634.1	Q2HWU5	531
459.	Eucarya	AGLU	GH13_40	*Kluyveromyces lactis*	CAB46746.1	Q9Y844	583
460.	Eucarya	AGLU	GH13_40	*Saccharomyces cerevisiae*	ADK27710.1	E1AFY6	589
461.	Eucarya	AGLU	GH13_40	*Pichia angusta*	AAF69018.2	Q9P8G8	564
462.	Bacteria	T6PH	GH13_29	*Lactobacillus acidophilus*	AAV42863.1	Q5FKB1	554
463.	Bacteria	T6PH	GH13_29	*Bacillus licheniformis*	AAU22384.1	Q65MI2	562
464.	Bacteria	T6PH	GH13_29	*Escherichia coli*	BAB38639.1	Q8XCE1	551
465.	Bacteria	AGLU	GH13_30	*Bifidobacterium adolescentis*	BAF39233.1	A1A0K0	590
466.	Bacteria	AGLU	GH13_30	*Thermobifida fusca*	AAZ54871.1	Q47RP6	544
467.	Bacteria	AGLU	GH13_30	*Arthrobacter globiformis*	BAI67603.1	D2YYD7	567
468.	Bacteria	OGLU	GH13_23	*Bacillus flavocaldarius*	BAB18518.1	Q9F237	529
469.	Bacteria	AGLU	GH13_23	*Halomonas* sp. H11	BAL49684.1	H3K096	538
470.	Bacteria	ATGS	GH13_23	*Xanthomonas campestris*	BAC87873.1	H3K096	538
471.	Eucarya	AGLU	GH13_17	*Anopheles gambiae*	CAA60857.1	Q17021	498
472.	Eucarya	SUH	GH13_17	*Bombyx mori*	BAP18683.1	A0A077JI83	606
473.	Eucarya	AGLU	GH13_17	*Apis mellifera*	BAA11466.1	Q17058	567
474.	Eucarya	rBAT	GH13_35	*Homo sapiens* (isoform A)	AAA35500.1	Q07837	685
475.	Eucarya	rBAT	GH13_35	*Ovis aries*	KAG5211578.1	A0A6P7DVK7	685
476.	Eucarya	rBAT	GH13_35	*Salmo salar*	ACN11390.1	C0HBH1	681
477.	Bacteria	AMS	GH13_4	*Deinococcus geothermalis*	ABF44874.1	Q1J0W0	650
478.	Bacteria	SUH	GH13_4	*Xanthomonas campestris*	AAM42629.1	Q8P5I2	637
479.	Bacteria	AMS	GH13_4	*Neisseria polysaccharea*	EFH23057.1	E2PEX2	636
480.	Bacteria	TSY	GH13_16	*Mycolicibacterium smegmatis*	ABK71531.1	A0R6E0	593
481.	Archaea	TSY	GH13_16	*Picrophilus torridus*	AAT42654.1	Q6L2Z7	558
482.	Bacteria	TSY	GH13_16	*Deinococcus radiodurans*	AAF11586.1	Q9RST7	552
483.	Bacteria	AGLU	GH13_44	Bifidobacteriaceae bacterium	RFT33048.1	A0A3E2CLU2	566
484.	Bacteria	HYPO	GH13_44	*Gardnerella vaginalis*	ADB14357.1	D2RCE7	566
485.	Bacteria	HYPO	GH13_44	*Alistipes shahii*	CBK63352.1	D4IK90	559
486.	Bacteria	AAMY	GH13_36	*Bacteroides thetaiotaomicron*	AAB42174.1	Q8A1G3	692
487.	Bacteria	AAMY	GH13_36	*Dictyoglomus thermophilum* (AmyC)	CAA34072.1	P14899	499
488.	Bacteria	AAMY	GH13_36	*Halothermothrix orenii* (AmyA)	ACL70223.1	B8CY54	515

[a] Sequences highlighted by yellow were selected for further phylogenetic analysis of reduced dataset.

[b] The individual enzymes are abbreviated as follows: CMD, cyclomaltodextrinase; HYPO, hypothetical enzyme; AAMY, α-amylase; NPUL, neopullulanase; CGT, cyclodextrin glucanotransferase; MGAA, maltogenic α-amylase; GPMT, α-1,4-glucan: phosphate α-maltosyltransferase; AMS, amylosucrase; SUH, sucrose hydrolase; M6H, maltohexaose-producing amylase; GBE, α-glucan branching enzyme; MOTH, maltooligosyltrehalosetrehalohydrolase; ISA, isoamylase; GDGE, glycogen-degrading enzyme; PUL, pullulanase; LDE, limit dextrinase; TSY, trehalose synthase; AGLU, α-glucosidase; SPH, sucrose phosphorylase; CMMH, cyclic maltosyl-maltose hydrolase; NSA, non-specified amylase; MGA, maltogenic amylase; APUL, amylopullulanase; AGS, α-1,3-glucan synthase; OGLU, oligo-1,6-glucosidase; ATGS, α-transglucosidase; GDE, glycogen debranching enzyme; MOTS, maltooligosyltrehalosetrehalosynthase; T6PH, trehalose-6-phosphate hydrolase; SIM, sucrose isomerase; DGLU, dextran glucosidase; 4F2hc, 4F2 heavy-chain antigen; rBAT, amino acid transport protein rBAT; M3H, maltotriose-producing amylase.

[c] The two sequences, No. 349 and No. 350, in the context of the present study, belong to the so-called “intermediary group without assignment to any GH13 subfamily so far.

Table S2. Results of the tertiary structure comparison of the GH13_48 maltogenic amylases from *T. neapolitana*, *L. plantarum* and the GH13_49 α-amylase from *H. japonica* with representatives of all other 47 well-established GH13 subfamilies.

				**MGA from *T. neapolitana* (4GKL)**	**MGA from *L. plantarum* (3DHU)**	**AAMY from *H. japonica* model (L8B068)**
**Subfamily**	**Enzyme**	**Source**	**PDB code**	**Cα; RMSD (Å)**	**Cα; RMSD (Å)**	**Cα; RMSD (Å)**
GH13_1	AAMY	*Aspergillus oryzae*	2TAA	183; 1.29	153; 1.34	168; 1.23
GH13_2	CGT	*Bacillus circulans*	1CDG	181; 1.24	178; 1.19	214; 1.12
GH13_3	GPMT	*Mycobacterium tuberculosis*	4U33	205; 1.16	194; 1.13	181; 1.1
GH13_4	AMS	*Deinococcus geothermalis*	3UCQ	163; 1.08	143; 1.10	213; 1.11
GH13_5	AAMY	*Bacillus amyloliquefaciens*	3BH4	130; 1.13	128; 1.12	157; 1.19
GH13_6	AAMY	*Hordeum vulgare*	1HT6	139; 1.17	139; 1.14	152; 1.15
GH13_7	AAMY	*Pyrococcus woesei*	3QGV	110; 1.15	95; 0.99	166; 1.11
GH13_8	GBE	*Homo sapiens*	4BZY	142; 1.05	130; 1.02	192; 1.05
GH13_9	GBE	*Cyanothece* sp. ATCC 51142	5GQU	143; 1.17	161; 1.17	183; 1.16
GH13_10	MOTH	*Deinococcus radiodurans*	2BHU	196; 1.08	173; 0.99	222; 1.09
GH13_11	ISA	*Sulfolobus solfataricus*	2VNC	140; 1.02	162; 1.11	151; 1.05
GH13_12	PUL	*Streptococcus pneumoniae*	2YA0	176; 1.18	189; 1.13	181; 1.17
GH13_13	LDE	*Hordeum vulgare*	4J3S	142; 1.2	146; 1.21	131; 1.22
GH13_14	PUL	*Anoxybacillus* sp. LM18-11	3WDH	147; 1.25	183; 1.2	188; 1.18
GH13_15	AAMY	*Tenebrio molitor*	1CLV	139; 1.15	129; 1.17	164; 1.16
GH13_16	TSY	*Mycolicibacterium smegmatis*	3ZO9	212; 1.08	177; 1.07	225; 1.1
GH13_17	SUH	*Bombyx mori*	6LGA	173; 1.05	160; 0.99	169; 1.1
GH13_18	SPH	*Bifidobacterium adolscentis*	1R7A	133; 1.36	103; 1.19	139; 1.2
GH13_19	AAMY	*Escherichia coli*	8IM8	166; 1.09	153; 1.06	177; 1.01
GH13_20	NPUL	*Thermoactinomyces vulgaris* TVA-II	1BVZ	187; 1.11	237; 1.15	229; 1.13
GH13_21	AAMY	*Thermoactinomyces vulgaris* TVA-I	1UH3	188; 1.12	163; 1.12	232; 1.08
GH13_22	AGS	*Schizosaccharomyces pombe*	model (Q9UUL4)	132; 1.25	127; 1.14	157; 1.1
GH13_23	ATGS	*Xanthomonas campestris*	6AAV	221; 1.15	221; 1.06	195; 1.07
GH13_24	AAMY	*Homo sapiens*	1HNY	109; 1.18	122; 1.16	140; 1.18

GH13_25	GDE	*Candida grablata*	5D06	66; 1.13	72; 1.34	26; 1.04
GH13_26	MOTS	*Sacharolobus shibatae*	5ZCR	69; 1.05	10; 1.07	114; 0.99
GH13_27	AAMY	*Aeromonas hydrophila*	model (P22630)	117; 1.05	102; 1.07	143; 1.12
GH13_28	AAMY	*Bacillus subtilis*	1BAG	123; 1.11	136; 1.06	110; 1.06
GH13_29	T6PH	*Bacillus licheniformis*	5BRP	191; 1.,04	205; 0.95	175; 1.06
GH13_30	AGLU	*Arthrobacter globiformis*	model (D2YYD7)	179; 0.95	165; 0.88	221; 0.96
GH13_31	OGLU	*Bacillus cereus*	1UOK	204; 1.19	196; 0.99	186; 1.08
GH13_32	AAMY	*Pseudoalteromonas haloplanktis*	1AQH	100; 1.04	116; 1.14	134; 1.11
GH13_33	TSY	*Pseudomonas stutzeri*	model (Q9LAS5)	127; 1.23	128; 1.2	130; 1.09
GH13_34	4F2hc	*Homo sapiens*	2DH2	165; 1.07	140; 1.2	156; 1.11
GH13_35	rBAT	*Homo sapiens*	6LI9	211; 1.11	171; 1.12	222; 1.05
GH13_36	AAMY	*Bacteroides thetaiotaomicron*	3K8K	200; 1.02	173; 0.97	227; 1.0
GH13_37	AAMY	uncultured bacterium	5H05	187; 1.16	181; 1.02	203; 1.07
GH13_38	AGLU	*Bacteroides_cellulosilyticus*	model (A0A0P0GJC2)	235; 1.09	176; 1.03	232; 1.02
GH13_39	APUL	*Thermoanaerobacter thermohydrosulfuricus*	model (P16950)	197; 1.14	172; 0.98	236; 1.0
GH13_40	AGLU	*Sacharomyces cerevisiae*	3A47	175; 1.09	170; 1.1	164; 0.95
GH13_41	AAMY	*Micrococcus* sp. 207	model (Q06812)	192; 1.14	173; 1.09	222; 1.06
GH13_42	M6H	*Microbacterium aurum*	model (A0A1B3IKE0)	159; 1.17	136; 1.1	172; 1.08
GH13_43	AAMY	*Haloarcula hispanica*	model (Q4A3E0)	123; 1.10	124; 1.12	163; 1.03
GH13_44	AGLU	Bifidobacteriaceae bacterium	model (A0A3E2CLU2)	208; 1.11	193; 0.99	220; 0.99
GH13_45	AAMY	*Geobacillus thermoleovorans*	4E2O	205; 1.04	176; 0.98	245; 1.09
GH13_46	CMD	*Flavobacterium* sp. No. 92	1H3G	217; 1.14	179; 1.1	224; 1.04
GH13_47	AAMY	*Bacteroides ovatus*	8DGE	177; 1.1	183; 1.1	153; 1.02
GH13_48	MGA	*Thermotoga neapolitana*	4GKL	-	283; 1.04	228; 1.05
GH13_48	MGA	*Lactoplantibacillus plantarum*	3DHU	-	-	216; 1.06
GH13_49	AAMY	*Haloarcula japonica* TR-1	model (L8B068)	-	-	-

Figure S1. Sequence alignment of the entire family GH13 with a focus on the two novel subfamilies GH13_48 and GH13_49. The alignment covers the catalytic TIM-barrel domain including the domain B spanning the segment from the beginning of the strand β2 to the end of the strand β8 (involving all seven CSRs) of 488 sequences of the present study (Table S1). Seven well-established CSRs of the family GH13 are boxed with black frames. The catalytic residues – aspartic acid, glutamic acid and aspartic acid – are located in CSR-II, CSR-III and CSR-IV, respectively. Identical and similar positions are signified, respectively, by asterisks and dots/semicolons under the alignment blocks. The colour code for the selected residues: W – yellow; F, Y – blue; V, L, I – green; D, E – red; R, K – cyan; H – brown; C – magenta; G, P – black. The labels of protein sources consist of the GenBank accession number and the name of the organism. In case of experimentally characterized enzymes, their abbreviation is also used (Table S1). The sequence order in the alignment (starting from the top) reflects their order in the evolutionary tree (Fig. S2) in the anticlockwise manner (starting from the first sequence in the blue cluster).

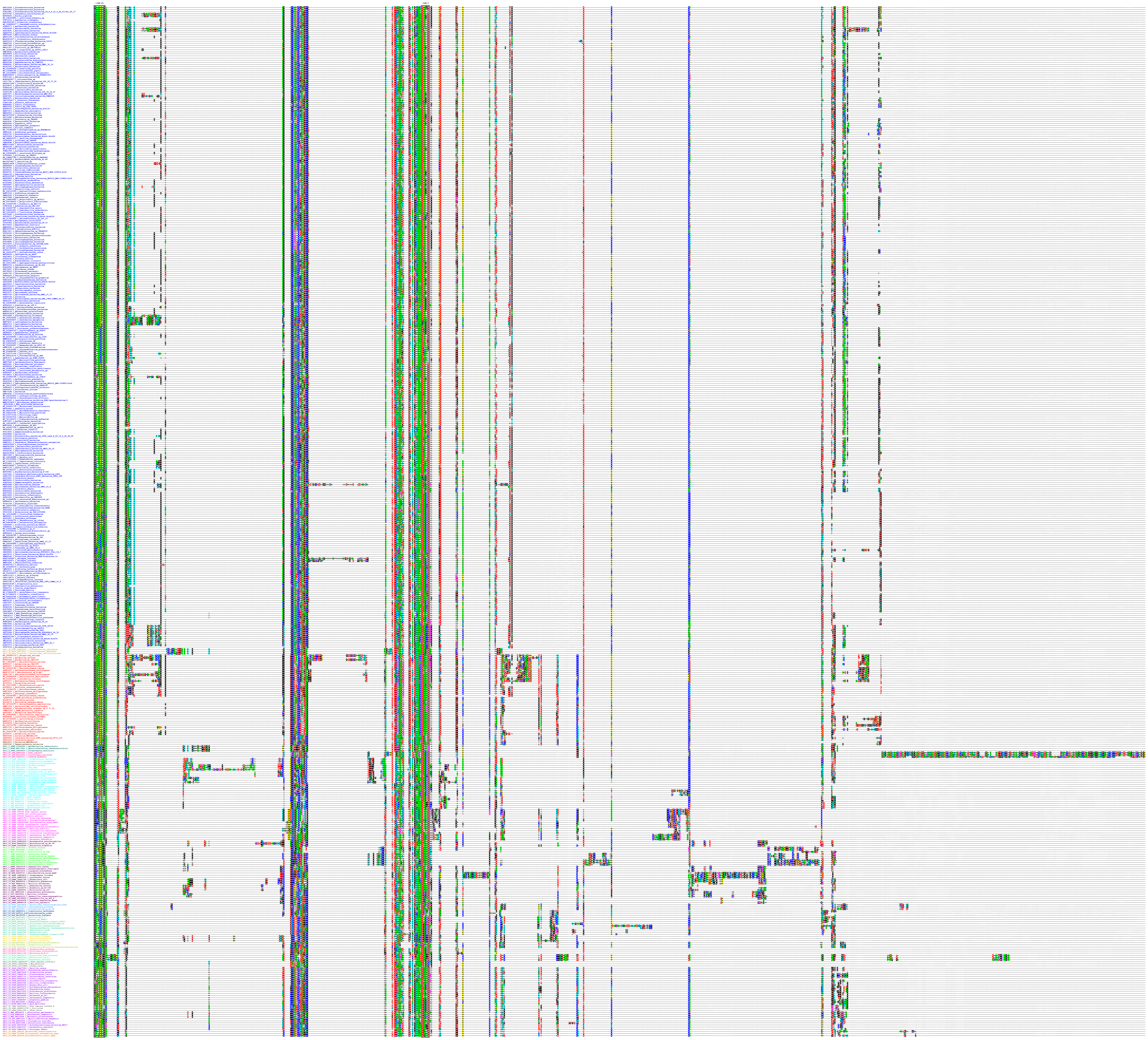

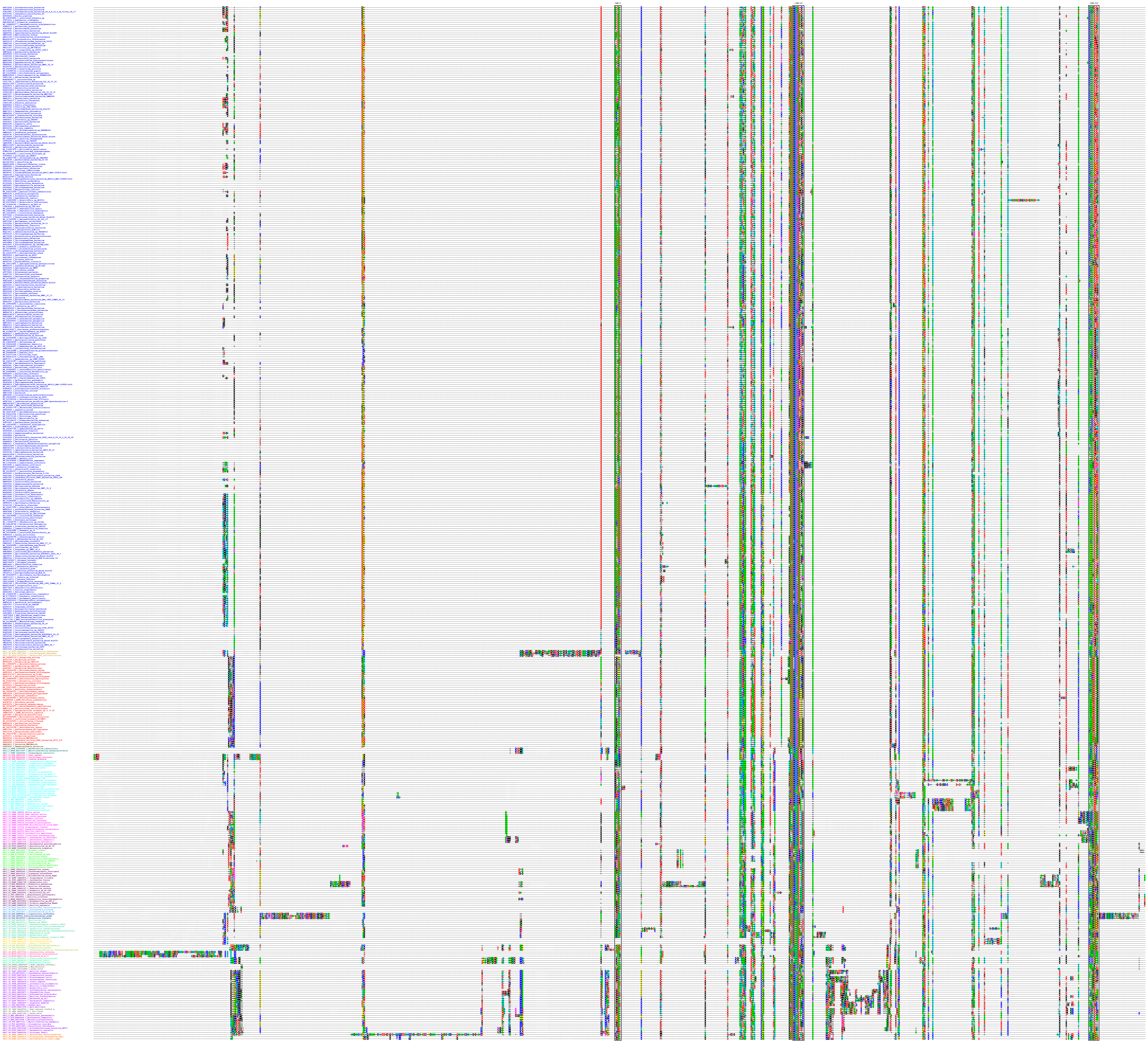

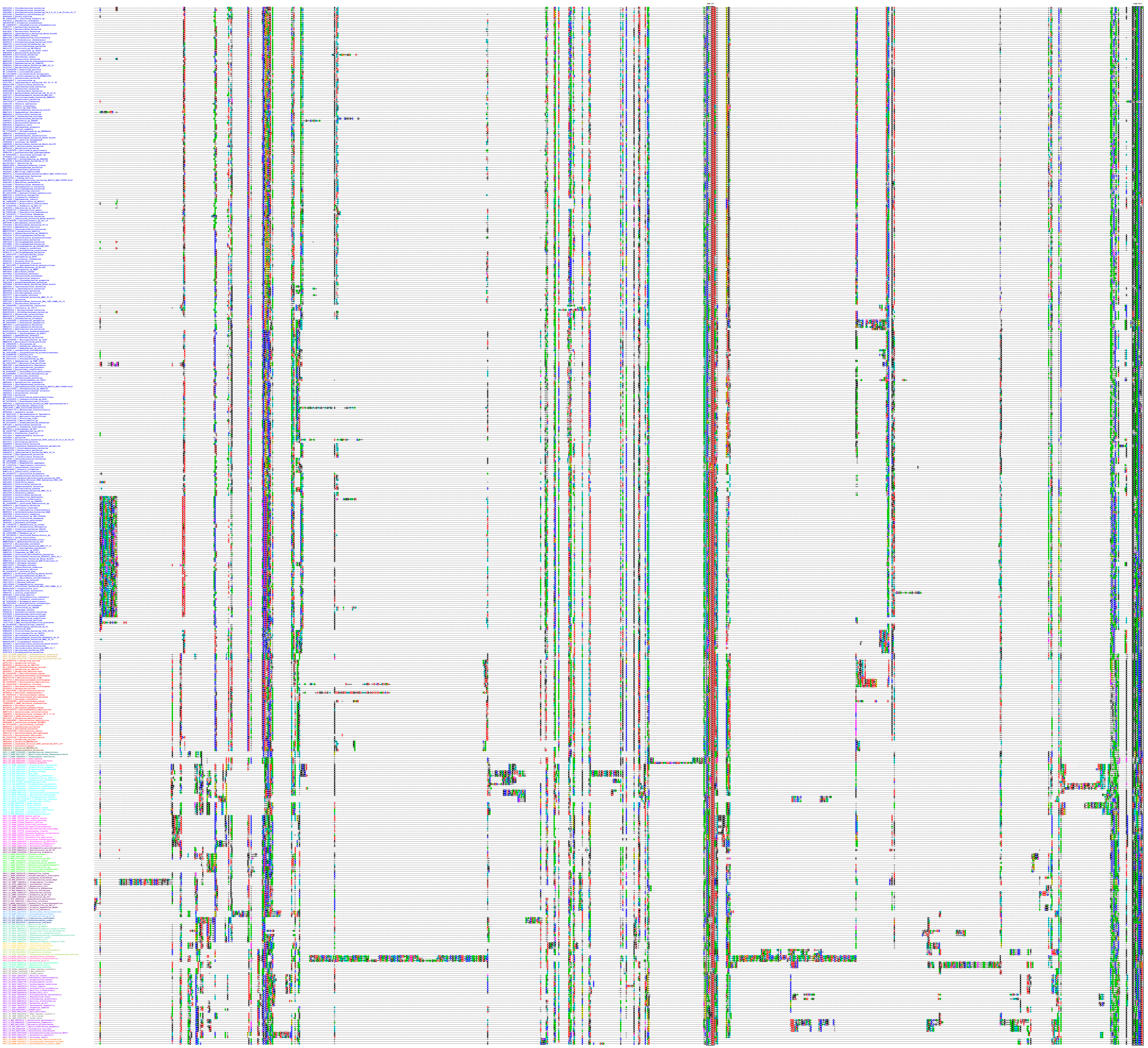

Figure S2. Evolutionary tree of the main α-amylase family GH13. The tree covers 488 sequences with a focus on the two novel closely related subfamilies GH13_48 and GH13_49 represented by the maltogenic amylase from *T. neapolitana* and the α-amylase from *H. japonica*, respectively (for details, see Table S1). The tree is based on the alignment (Fig. S1). The labels of protein sources consist of the GenBank accession number and the name of the organism. In case of experimentally characterized enzymes, their abbreviation is also used (Table S1). Characterized members of the two novel subfamilies are labelled by an asterisk. The same tree in a simplified version without the leaves, i.e. without sequence description, is presented in Figure 1.

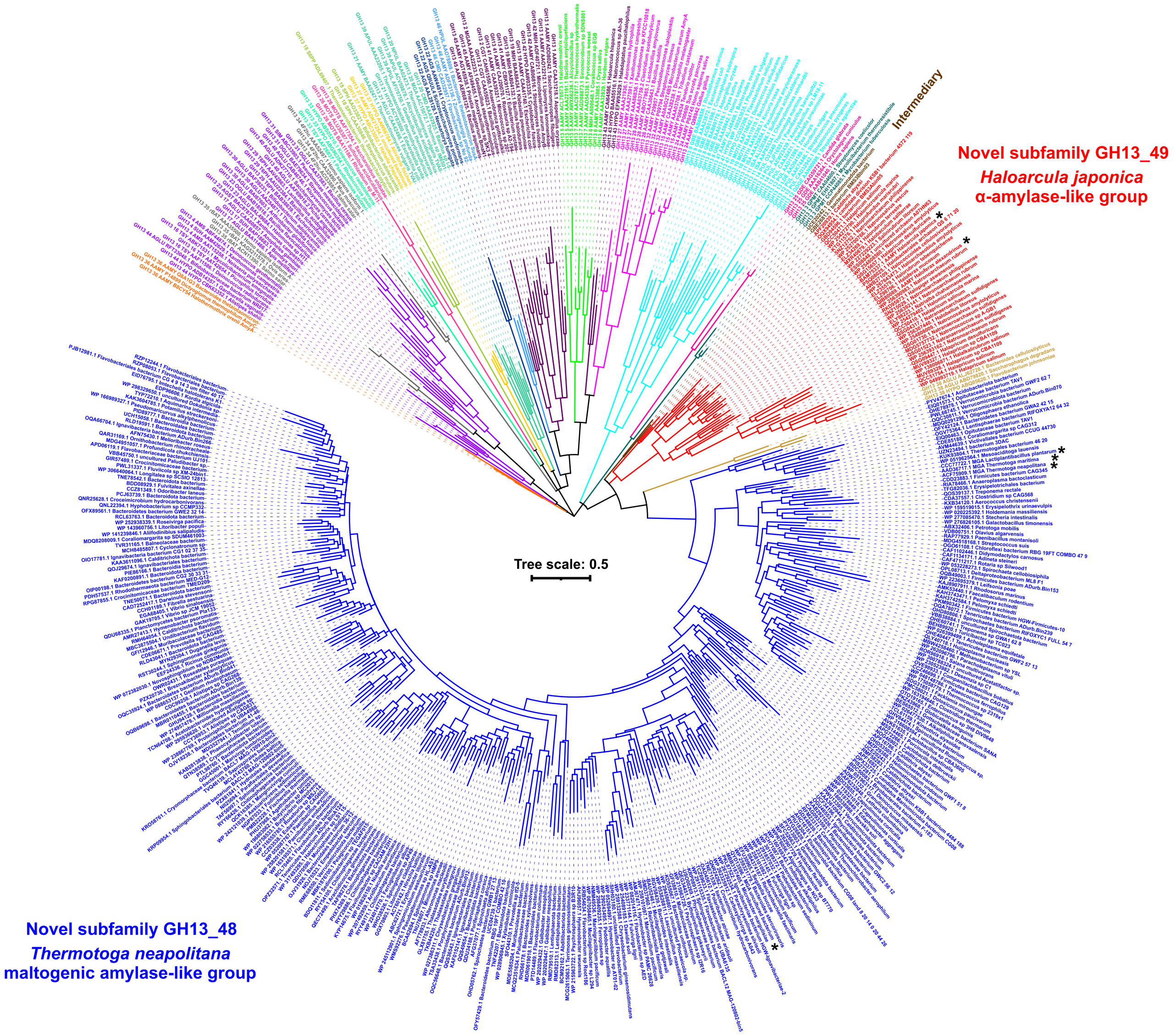

Figure S3. Sequence alignment of a reduced sample of 38 full-length sequences with a focus on the two novel subfamilies GH13_48 and GH13_49. Seven well-established CSRs of the family GH13 are boxed with black frames. The catalytic residues – aspartic acid, glutamic acid and aspartic acid – are located in CSR-II, CSR-III and CSR-IV, respectively. Identical and similar positions are signified, respectively, by asterisks and dots/semicolons under the alignment blocks. The colour code for the selected residues: W – yellow; F, Y – blue; V, L, I – green; D, E – red; R, K – cyan; H – brown; C – magenta; G, P – black. The labels of protein sources consist of the GenBank accession number and the name of the organism. In case of experimentally characterized enzymes, their abbreviation is also used (Table S1). The sequence order in the alignment (starting from the top) reflects their order in the evolutionary tree (Fig. 2) in the anticlockwise manner (starting from the first sequence in the red cluster).

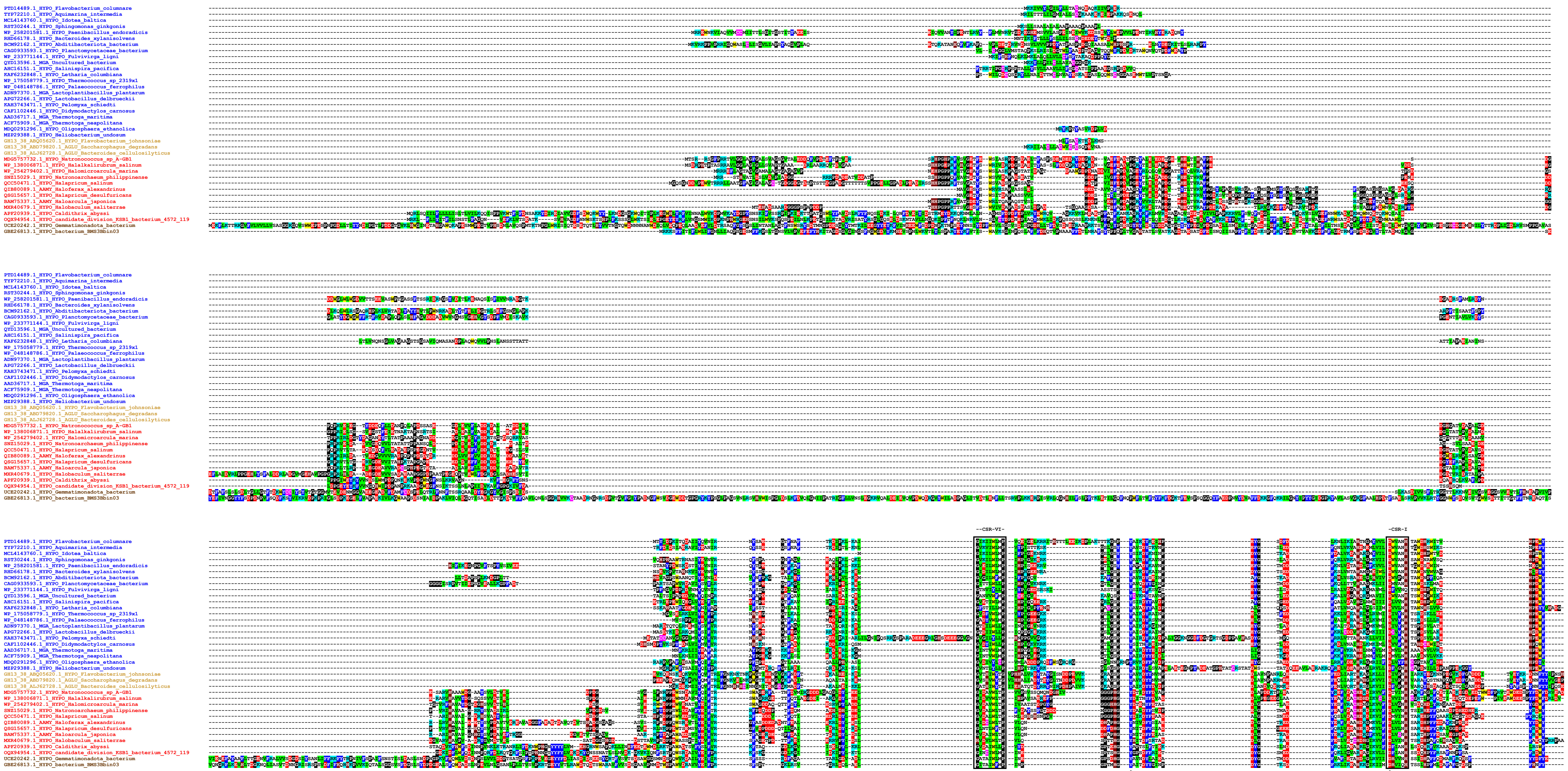
--CSR-VI-
-CSR-I

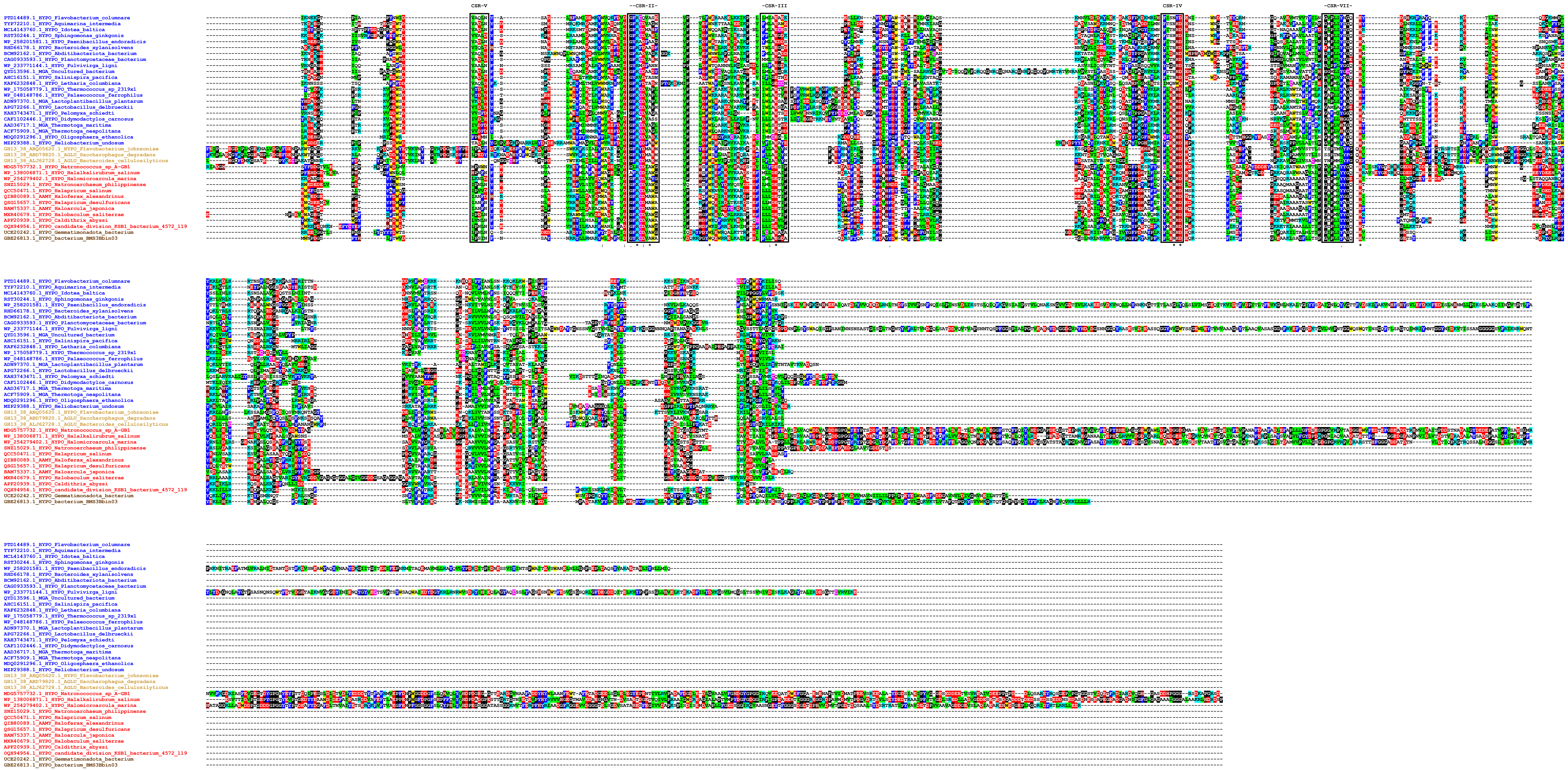

CSR-V
--CSR-II-
-CSR-III
CSR-IV
-CSR-VII-
PTD14489.1_HYPO_Flavobacterium_columnare
TYP72210.1_HYPO_Aquimarina_intermedia
MCL4143760.1_HYPO_Idotea_baltica
RST30244.1_HYPO_Sphingomonas_ginkgonis
WP_258201581.1_HYPO_Paenibacillus_endoradicis
RHD66178.1_HYPO_Bacteroides_xylanisolvens
BCM92162.1_HYPO_Abditibacteriota_bacterium
CAG0933593.1_HYPO_Planctomycetaceae_bacterium
WP_233771144.1_HYPO_Fulvivirga_ligni
QYD13596.1_MGA_Uncultured_bacterium
AHC16151.1_HYPO_Salinispira_pacifica
KAF6232848.1_HYPO_Letharia_columbiana
WP_175058779.1_HYPO_Thermococcus_sp_2319x1
WP_048148786.1_HYPO_Palaeococcus_ferrophilus
ADN97370.1_MGA_Lactoplantibacillus_plantarum
APG72266.1_HYPO_Lactobacillus_delbrueckii
KAH3743471.1_HYPO_Pelomyxa_schiedti
CAF1102446.1_HYPO_Didymodactylos_carnosus
AAD36717.1_MGA_Thermotoga_maritima
ACF75909.1_MGA_Thermotoga_neapolitana
MDQ0291296.1_HYPO_Oligosphaera_ethanolica
MZP29388.1_HYPO_Heliobacterium_undosum
GH13_38_ABQ05620.1_HYPO_Flavobacterium_johnsoniae
GH13_38_ABD79820.1_AGLU_Saccharophagus_degradans
GH13_38_ALJ62728.1_AGLU_Bacteroides_cellulosilyticus
MDG5757732.1_HYPO_Natronococcus_sp_A-GB1
WP_138006871.1_HYPO_Halalkalirubrum_salinum
WP_254279402.1_HYPO_Halomicroarcula_marina
SNZ15029.1_HYPO_Natronoarchaeum_philippinense
QCC50471.1_HYPO_Halapricum_salinum
QIB80089.1_AAMY_Haloferax_alexandrinus
QSG15657.1_HYPO_Halapricum_desulfuricans
BAM75337.1_AAMY_Haloarcula_japonica
MXR40679.1_HYPO_Halobaculum_saliterrae
APF20939.1_HYPO_Caldithrix_abyssi
OQX94954.1_HYPO_candidate_division_KSB1_bacterium_4572_119
UCE20242.1_HYPO_Gemmatimonadota_bacterium
GBE26813.1_HYPO_bacterium_BMS3Bbin03
